# Evaluating the Longitudinal Item and Category Stability of the SF-36 Full and Summary Scales Using Rasch Analysis

**DOI:** 10.1155/2018/1013453

**Published:** 2018-11-04

**Authors:** Reinie Cordier, Ted Brown, Lindy Clemson, Julie Byles

**Affiliations:** ^1^Curtin University, School of Occupational Therapy, Social Work and Speech Pathology, Bentley, Australia; ^2^Monash University, Occupational Therapy Department, Melbourne, Victoria, Australia; ^3^The University of Sydney, Faculty of Health Sciences, Sydney, New South Wales, Australia; ^4^The University of Newcastle, School of Medicine and Public Health, Callaghan, New South Wales, Australia

## Abstract

**Introduction:**

The Medical Outcome Study Short Form 36 (SF-36) is widely used for measuring Health-Related Quality of Life (HRQoL) and has undergone rigorous psychometric evaluation using Classic Test Theory (CTT). However, Item Response Theory-based evaluation of the SF-36 has been limited with an overwhelming focus on individual scales and cross-sectional data.

**Purpose:**

This study aimed to examine the longitudinal item and category stability of the SF-36 using Rasch analysis.

**Method:**

Using data from the 1921-1926 cohort of the Australian Longitudinal Study on Women's Health, responses of the SF-36 from six waves of data collection were analysed. Rasch analysis using Winsteps version 3.92.0 was performed on all 36 items of the SF-36 and items that constitute the physical health and mental health scales.

**Results:**

Rasch analysis revealed issues with the SF-36 not detected using classical methods. Redundancy was seen for items on the total measure and both scales across all waves of data. Person separation indexes indicate that the measure lacks sensitivity to discriminate between high and low performances in this sample. The presence of Differential Item Functioning suggests that responses to items were influenced by locality and marital status.

**Conclusion:**

Previous evaluations of the SF-36 have relied on cross-sectional data; however, the findings of the current study demonstrate the longitudinal efficacy of the measure. Application of the Rasch Measurement Model indicated issues with internal consistency, generalisability, and sensitivity when the measure was evaluated as a whole and as both physical and mental health summary scales. Implications for future research are discussed.

## 1. Introduction

To be deemed effective and useful, health measures must fulfil several requirements including validity, reliability, interpretability, and responsiveness to change [1]. Measurement invariance is another important characteristic, ensuring that the same construct is being consistently measured across different populations and settings, and over time. Considerations of measurement invariance are important for longitudinal studies that seek to gauge change in a construct, across a broad population and over time. When studies involve an older population, measurements may be vulnerable to instability as the participants age, their living circumstances may change, and their physical and cognitive abilities may decline [2, 3].

The Medical Outcome Study Short Form 36 (SF-36) is one of the most commonly used questionnaires for monitoring Health-Related Quality of Life (HRQoL) across a multitude of populations and settings, including client groups and healthy populations [4–10]. HRQoL refers to aspects of quality of life that are impacted by an individual's mental and physical health [11].

Development of the SF-36 came about following difficulties during the Health Insurance Experiment (HIE), whereby the completion of a lengthy health survey was refused by participants [9]. In response to this need, Ware et al. [9] constructed a health survey that was both comprehensive and relatively short. The initial survey, the SF-18, comprised of 18 items measuring physical functioning, role limitations relating to poor health, mental health and health perceptions [9]. Subsequently, additional items have been added to create the 20-item SF-20 version, and 36-item SF-36 version which is now the most commonly used.

The SF-36 measures eight key health concepts: (1) physical functioning (PF); (2) role limitations due to physical health problems (RL-P); (3) bodily pain (BP); (4) general health (GH); (5) vitality (V); (6) social functioning (SF); (7) role limitations due to emotional problems (RL-E); and (8) mental health (MH) [9]. From the eight scales, the survey generates overall physical and mental health component summary scores. Both summary measures include scores from all eight subscales; however particular correlations are present; the physical functioning, role limitations-physical, and bodily pain scales should correlate highest with the physical component score (PCS) and lowest with the mental component score (MCS) [12]. The mental health, role limitations-emotional, and social functioning scales should correlate highest with the MCS and lowest with PCS, with the remaining general health and vitality scales found to correlate moderately with both the PCS and MCS [12]. Summary score results can be compared with gender and age-group norms derived from the general population, e.g., United States population norms [12].

The SF-36 is now widely used for both research and clinical purposes and has undergone rigorous psychometric evaluation nationally and internationally using Classic Test Theory (CTT) [6, 7, 9, 10]. CTT seeks to determine the reliability of a whole instrument through evaluating the degree of variance in terms of the ratio between true and observed scores. Therefore observed results are the product of the respondent's “true score,” in combination with error [13].

A relatively new approach to psychometric test design is Item Response Theory (IRT; Edelen and Reeve) [14]. IRT models are typically considered to be unidimensional, assessing instrument reliability at item-level rather than instrument-level, by determining the unique contribution of each item to the construct or trait being measured. IRT considers the importance of participants' responses, whereby the probability of their answering a particular item correctly is based on their responses to other items of greater or lesser levels of difficulty or challenge [14]. Within IRT, the Rasch Measurement Model (RMM) is the most frequently applied IRT approach to investigating the unidimensionality of items that make up scales and to determining if responses are indeed measuring a single dimension only, through the examination of item fit statistics [15].

### 1.1. Application of IRT/RMM to the SF-36

Under the assumptions of an IRT model, instruments deemed reliable should meet the following properties: unidimensionality, hierarchical ordering of items, and reproducibility of scale items across client populations [16].* Unidimensionality* assumes that a collection of items represent and assess a single construct, that is, fit a single one dimensional model [16].* Item hierarchy* refers to a hypothesised continuum along which instrument items should progress in difficulty from easier to more challenging to answer. In other words, the probability of answering the more difficult items is higher for those individuals with higher levels of the latent trait being measured, while those with lower levels of the trait have a lower probability of answering items at the upper end [16].* Reproducibility* relates to item hierarchy whereby item order and calibrations along the continuum are seen to remain relatively stable or constant across different groups of assessment respondents and assessment occasions [16]. Item reproducibility or stability is considered essential to the ability to accurately measure between-group differences and within-group changes over time [16].

IRT-based evaluation of the SF-36 has overwhelmingly focused on individual scales, particularly the Physical Functioning-10 subscale, with only some studies having examined particular psychometric properties of the SF-36 as a whole instrument or by component summary scores [5, 6, 12, 16, 17].

### 1.2. Unidimensionality and Item Fit

Only a few analyses have investigated the model-fit of the SF-36 as a whole. A prospective cohort study, involving a sample of 583 participants who were opioid-dependent, assessed item-model fit and latent trait factors for the eight SF-36 subscales and for the whole instrument [6]. The RMM reliability estimates of all eight SF-36 subscales (including a revised PF-10 subscale) established that each measured a single latent trait [6]. Investigation of the dimensional structure of the instrument as a whole confirmed the presence of an eight-factor model; that is, the SF-36 measured eight distinct latent traits [6].

Analysis confirming a two-factor structure, reflecting the SF-36 physical and mental health components, has also been conducted using principal component analysis, with the physical and mental health domains accounting for 70% of the total variance across both standard and acute forms [12]. A single-administration survey with a general U.S.A. population sample (*n* = 634) evaluated the item-fit of the SF-36 physical and mental HRQoL domains using RMM modelling [5]. In this analysis, eight items in the physical domain had disordered thresholds, whereby a person responding to higher or lower levels of a categorical scale did not necessarily possess higher or lower levels of the trait that was being assessed [5]. The authors suggested collapsing some category options to overcome this issue [5]. In terms of the HRQoL domains' unidimensionality, the mental health items were seen to fit RMM expectations, whereas the physical domain required discarding of the seven misfitting items to produce a 14-item domain that met RMM requirements. Survey data for of 395 Taiwanese patients with chronic lung disease were analysed to conduct similar assessments of the SF-36 mental and physical health domains, with the authors concluding that each domain was unidimensional [7].

Differential item functioning (DIF) analysis using IRT-based techniques has also been undertaken with the SF-36. DIF refers to the unequal endorsement of instrument items by respondents of different groups, given that the items intend to measure the same latent trait [10]. The presence of DIF undermines instrument construct validity and may compromise the ability to compare instrument scores across different groups of respondents [10]. Yu et al. [10] utilised the multiple-indicator, multiple-causes (MIMIC) technique, and an IRT-based methodology to detect if DIF existed in the SF-36 physical and mental health domains. Data were extracted from the 1994-95 cohort of the Southern California Kaiser Permanente database (*n* = 7,538), which evaluated the health outcomes of patients receiving pharmacist consultations. DIF across SF-36 physical and mental health domains was analysed in relation to the presence of five key disease types: hypertension, rheumatic conditions, respiratory diseases, depression, and diabetes. Results indicated the presence of statistically significant DIF for a total of five items, both physical and mental health-based, for the hypertension, respiratory, and diabetes groups, respectively [10]. The authors concluded that the presence of DIF for only five of 36 items did not warrant significant concern regarding the overall construct validity of the SF-36; however, they cautioned regarding the use of the SF-36 in comparing groups based on hypertension in particular, who returned DIF effect for two items in the physical health domain [10].

### 1.3. Cross Cultural Item Response Patterns

Rasch modelling has also been applied to translated versions of the SF-36 to examine its cross-cultural validation. An assessment of the appropriateness of a Korean version of the SF-36 with 510 elderly Korean adults was conducted using the RMM [17]. The authors verified the presence of unidimensionality in the instrument and determined through step calibration that the response options of three- and five-point scales for items were appropriate for this population [17]. Goodness-of-fit statistics however determined that nine items across the instrument were not appropriate for this population, in terms of being incongruent with other items, having significant overlap with other items, or creating confusion due to misinterpretation of the meaning of items [17].

### 1.4. Item Stability

While item-model fit and determination of the presence of DIF are important, these properties can mean very little if item responses are inconsistent or changeable over time. Evaluation of the stability of item responses is important to determining the rigour of an instrument. Most IRT evaluations of SF-36 data have been cross-sectional and therefore stability of item response has not been evaluated [5–7, 10, 17]. Two studies assessed performance across repeated administrations, following pre-post designs [18, 19]. Martin et al. [18] utilised the SF-36 as one of three evaluation tools pre- and posttreatments for rheumatoid arthritis (*n* = 339), but with the aim to compare measurement properties of these tools and determine sensitivity to change rather than stability. IRT analysis of the PF-10 revealed weaknesses in sensitivity to treatment response at 6 and 12 months, with authors suggesting construction of a more comprehensive measure. McHorney et al. [19] compared IRT and Likert scoring method of the SF-36 Physical Functioing-10 scale, using a pre-post design. The findings showed apparent differences in patients with very high and low physical functioning, suggesting that Rasch model of scoring may have important implications for clinical interpretations of the scale [19].

Only one longitudinal study has evaluated properties of the SF-36 using IRT methodologies. The first administration of the standardised SF-36 was conducted as part of a four-year longitudinal Medical Outcomes Study of patients (*N* = 3,445) with chronic medical and psychiatric conditions [16]. Examination of the reproducibility of the item calibrations of the Physical Functioning-10 scale was conducted, from baseline to two years [16]. A high degree of consistency in item calibration between the two time points was found, both in order and magnitude [16]. However, this longitudinal study only evaluated the stability and structural validity of the Physical Functioing-10 scale using IRT. The stability of the remaining SF-36 subscales, the physical and mental health domains, and the measure as a whole over time has not been examined using IRT to date.

A lack of evaluation regarding the performance of the SF-36 over time presents a significant gap in the literature, with unanswered questions about its measurement stability. It is vital that the long-term reliability of the SF-36 is examined, to determine its true suitability for inclusion in large-scale longitudinal studies tracking participants, particularly as they age over extended periods of time. This study therefore seeks to use an IRT-based methodology to evaluate the item stability of the SF-36 total and component summaries in a large, longitudinal data set. The following questions guided this research:

1. Is there disordering or dysfunction within the SF-36 items against the construct being measured?
2. Do the SF-36 items have a consistent hierarchy of difficulty and good distribution across all waves of a longitudinal survey?
3. Is the SF-36 differentiating discreet subgroups of people reliably (e.g., urban vs. regional)?
4. Does the SF-36 measure one or more constructs?
5. Were all items in the SF-36 instrument used by all participant subgroups in the same way?

## 2. Methods

Data were from an Australian prospective, population-based survey. The Australian Longitudinal Study on Women's Health (ALSWH) aims to assess physical and emotional health, use of health services, health risk factors and behaviours, life stages, and demographic characteristics. The ALSWH is conducted by researchers from the University of Newcastle and the University of Queensland and is funded by the Australian Government Department of Health. The study commenced in 1996 and has been running for over 20 years.

### 2.1. Participants

Three cohorts of women born in 1973-78 (aged 18-23 in 1996), 1946-51 (aged 45-50), and 1921-26 (aged 70-75) were randomly selected from the Medicare database, which includes all Australian citizens and permanent residents. Women living in regional and remote areas were sampled at twice the rate of women living in urban areas in order to allow for meaningful statistical comparisons between urban and country-dwelling women.

Over 40,000 respondents initially responded to the baseline postal survey in 1996 with response rates across the three age groups ranging between 37% and 52% [20]. Although some immigrant groups were underrepresented and tertiary educated women were overrepresented, the responding samples were considered to be “reasonably representative” of the Australian female adult population following a comparison to census data [21]. Each cohort has since been surveyed every three years on a rolling basis, commencing with the 1946-51 cohort in 2018, the 1921-26 cohort in 1999, and the 1973-78 cohort in 2000. Only data from the 12,432 respondents in the 1921-26 cohort were analysed in the current study. At the commencement of the longitudinal survey, these women were aged 70-75 years, and at the time of survey six, they were aged in their early nineties (*N* = 4,055), with most attrition being due to death (*N* = 5,273).

A study analysed potential biases introduced through the attrition of participants from this cohort between survey one and survey five [22]. Nondeath attrition was related to having less education, not being born in Australia, being a current smoker, and having poorer health in this cohort. Analysis comparing the survey population to the Australian Census data collected over the same time period showed an increase in the underrepresentation of women from non-English speaking backgrounds and an increase in the overrepresentation of current and ex-smokers. Differences between the study population and the national population were considered to have changed “only slightly” between survey one and survey five.

### 2.2. Instrument

The SF-36 HRQoL scale is included in each survey. At baseline in 1996, mean scores for the 1921-26 cohort were lower than for other cohorts for the physical health subscales (PF, RP, and BP) and higher than for other cohorts for the mental health subscales (MH, RE, and BP) [23]. Over time, mean PF scores scale have declined, but with significant variation across different subgroups within the cohort [24]. Mean MH scores have remained relatively stable [25].

### 2.3. Data Analysis

A two-stepped approach was taken to evaluate the reliability and validity of the SF-36. Across surveys one to six. First, Rasch analyses using Winsteps version 3.92.0 [26], with the joint maximum likelihood estimation method [27] were performed on all 36 items for each of the six waves of data collection and then on the items that constitute the physical health scales (PF 10-items, RP 4 items, BP 2 items, and GH 5 items), the mental health scales (V, SF 2 items, RE 3 items, and MH 5 items) and the item measuring health transition for each wave of data. The RMM was adopted for the data analysis since the 6-point response Likert scale was invariant across all the 36 items. The RMM adopts a “the data fit the model” approach. “The empirical data must meet the prior requirements of Rasch model in order to achieve objective measurement” [28, p. 65]. Several criteria including item infit and outfit statistics, reliability measures, rating scale functioning, and differential item functioning (DIF) were used to investigate the quality of the SF-36 total scale, physical health scale, and mental health scale. Item fit statistics indicate the extent to which the data match the expectations of the RMM. Outfit and Infit mean square (MNSQ) as well as their standardized forms (ZSTD) are used.

#### 2.3.1. Is There Disordering or Dysfunction within the SF-36 Items against the Construct Being Measured? Response Scale

Category and step (threshold) disordering of the response scale was examined. To determine whether the rating response scales were being used in the expected manner, the rate at which average measure scores (frequency endorsed) increased in relation to category increases was examined for even distribution. A uniform category distribution is achieved when average measure scores increase monotonically as the category increases. If categories are poorly defined or items are included that do not fit the construct, then non-uniformity occurs. Fit mean squares (MNSQ) below 0.7 or above 1.4 indicate a category misfit. When disordered categories are measured then a consideration should be made to collapse it with an adjacent category [29].

The distance between categories is indicated by Andrich thresholds, or step calibrations. If there is no overlap, then categories should progress monotonically. Disordered steps indicate that the category defines only a narrow definition of the variable, rather than a problem with the sequencing of category definitions. An increase of at least 1.0 logit indicates distinct average measure categories on a 5-category scale, and gaps in the variable are indicated by an increase of >0.5 logits [30].

#### 2.3.2. Do the SF-36 Items Have a Consistent Hierarchy and Good Distribution across All Waves? Person and Item Fit Statistics

Misfitting items and the pattern of responses for each survey respondent were identified using fit statistics. These are used to determine whether an instrument is a valid measure of the construct it claims to measure. Fit statistics, reported as log odd units (logits), will be examined to determine whether the items contribute to the measurement a single construct, and the reliability of any one person's responses. The item constructs reviewed in this study are health related quality of life as a whole, as well as quality of life related to physical health and mental health. Two unstandardized statistics, MNSQ and Z-Standard (Z-STD), were used to measure item and person infit and outfit. MNSQ values for infit and outfit should have a value close to 1.0 to fit the model for rating scales, but values within the range of 0.7-1.4 are considered acceptable [15]. The model is degraded by underfit (i.e., values > 1.0), indicating the possibility for other sources of variance in the model and further investigation is required to determine the reason for the underfit. Conversely, overfit (values < 1.0) does not always degrade the model and could result in a misinterpretation that the model worked better than expected [15]. Z-STD values for outfit are expected to reach 0. If a value exceeds ±2, it is deemed to fall outside of the predicted model [15].

The person reliability statistic is equivalent to Cronbach's alpha used in CTT and indicates a measure's internal consistency (the relatedness amongst items) [15]. When person reliability values are low (i.e., < 0.8), the implications are twofold: (1) an instrument may not be sensitive enough to distinguish between high and low performers and more items are required; or (2) there were not enough persons in the sample with both high and low extreme values (a narrow range of person measures).

Person separation (if the outlying measures are accidental) and person separation index (PSI)/strata (if the outlying measures represent true performances; 4*∗*person separation +1/3) are used to classify people. Person separation reports whether the test separates the sample into enough levels with reliability of 0.5 separating into only one or two levels. Low person separation suggests that the instrument is not sensitive enough to separate high and low performers, 0.8 indicating separation into 2-3 levels and 0.9 indicating separation into 3 or 4 levels [29]. PSI/strata of 3 are needed to consistently identify three different levels of performance (i.e., the minimum level required to attain a reliability of 0.9). Item reliability verifies item hierarchy with <3 levels (high, medium, and low) with item reliability < 0.9 indicating the sample is too small to confirm the construct validity (item difficulty) of the instrument.

#### 2.3.3. Does the SF-36 Measure One or More Constructs? Dimensionality of the Scale

Dimensionality is tested by the following: (a) finding potentially problematic items by checking negative point-biserial correlations; (b) identifying misfitting persons or items using Rasch fit statistics; and (c) conducting Rasch factor analysis using principal components analysis (PCA) of the standardised residuals [31]. PCA of residuals checks that there are no further principal components (dimensions) after the intended or Rasch dimension is removed. No further dimensions are indicated if the residuals for pairs of items are uncorrelated and normally distributed. The criteria for determining the presences of further dimensions in the residuals were as follows: (1) >60% of the variance is explained by the Rasch factor; (2) an eigenvalue of <3 on first contrast; and (3) variance explained by the first contrast is <10% [32].

The person-item dimensionality map provides a schematic representation of how person abilities and item difficulties are distributed using a logit scale. Items that represent similar difficulty will occupy the same place on the logit scale. If a person is represented on the logit scale with no corresponding item, then there are gaps in the item difficulty continuum. Another indicator of overall distribution is the person measure score. If people in the sample are more able than the most difficult item on a scale, then the person measure score location will be lower than the centralised item mean measure score (i.e., <50). If people in the samples are less able than the items on a scale, then the mean person location will be higher (i.e. >50).

#### 2.3.4. Were All Items in the SF-36 Instrument Used by All Groups in the Same Way? Differential Item Analysis

A differential item analysis (DIF) was performed to investigate whether items in the instrument were used by all groups in the same way. DIF is noticeable when a response to an item is influenced by a characteristic of the respondent other than their ability on the underlying trait. For DIF analysis, the sample was categorised by marital status (single, widowed, divorced, married, de facto, and other) and location (urban vs. regional). In determining DIF when comparing two groups (i.e., urban and regional) the hypothesis “this item has the same difficulty for two groups” is used. The difference in the difficulty of the item between the two groups, indicated by the DIF contrast, should be at least 0.5 logits with a* p*-value < 0.05 for DIF to be noticeable. In determining DIF when comparing more than two groups (i.e., marital status) the hypothesis “this item has no overall DIF across all groups” is used. DIF is then determined using the chi-square statistic and* p*-value < 0.05 [29].

## 3. Results

SF-36 data were gathered over six waves: Wave 1,* N* = 12,077; Wave 2,* N* = 10,411; Wave 3,* N* = 8,577; Wave 4,* N* = 7,112, Wave 5,* N* = 5,534; and Wave 6,* N* = 4,032. The sample size decreased with each subsequent phase of data collection as participants died or were lost to follow-up.

### 3.1. SF36 Total Scale Rasch Analysis for Six Waves of Data Collection

Total Rasch scale item statistics for six waves of data collection are shown in Table 1. When all 36 SF-36 items were calibrated using the RMM for the six waves of data collection, MNSQ infit statistics ranged from 0.13 to 2.43 and outfit statistics ranging from 0.22 to 2.64 (see Table 2). The mean item measure was 0.00 logits (SD = 1.12). With respect to logit measures, there was a broad range, the lowest value being –3.01 and the highest value being +2.31. This resulted in an average item separation index of 77.98 and an average item reliability of 1.00 over the six waves (see Table 3).

The SF-36 total scale person-item map in Supplemental Figure 1 shows evidence of consistent hierarchical ordering of the SF-36 total scale items. Items which were less difficult are located at the bottom of the person-item map while more difficult items are located at the top of the map. The figure also shows that while each of the waves had a reasonable distribution of items in relation to item difficulty, several of the SF-36 total scale items have the same level of difficulty.

Rasch analysis reports the calibrations of the five thresholds (for the six-category rating scale) increase monotonically from -3.15, -1.36, -.25, .48, 1.31, and 2.82 for wave one and -2.96, -1.30, -.31, .42, 1.29, and 2.78 for wave six.

The average person measure was 0.75 logits (SD = 0.23) over the six waves of data collection (see Table 3). The mean person separation was 0.73 with a mean reliability of 0.35 (see Table 3). When examining the overall RMM output of the SF-36 total scale, the average person measure (0.75 logits) was higher than the average item measure (0.00 logits). The range of logit values for items was from +1 to -3 logits. The person reliability was 0.35 and item reliability was 1.00. This places the item reliability for the SF-36 total scale in the acceptable range and the person reliability correlation in the unacceptable range.

The separation index for items was greater than 2.0 indicating adequate separation of the items on the construct being measured. However, the separation index for persons was less than 2.0 indicating inadequate separation of participants on the construct.

Item fit to the unidimensionality requirement of the RMM was also examined. Eleven out of the 36 items were found to have MNSQ infit and outfit statistics inside the 0.70 to 1.30 range and/or a z-score that fell inside the +2 to -2 range. Specifically, items CH01:Q1, PF01:Q3A, PF04:Q3D, PF06:Q3F, PF07:Q3G, MH04:Q9F, VT03:Q9G, VT04:Q9I, SF02:Q10, CH02:Q11A, and GH04:Q11C met the RMM requirements (see Table 2). In other words, only 30.6% (i.e., 11 of 36) of the 36 SF-36 total scale items met the RMM requirements. The following items had an Infit MnSq statistic that was less than 0.70: HT:Q2, PF02:Q3B, PF03:Q3C, PF05:Q3E, PF08:Q3H, PF09:Q3I, PF10:Q3J, RP01:Q4A, RP02:Q4B, RP03:Q4C, RP04:Q4D, RE01:Q5A, RE02:Q5B, and RE03:Q5C. The following items had an Infit MNSQ statistic that was greater than 1.30: FO01:Q6, BP01:Q7, BP02:Q8, VT01:Q9A, MH01:Q9B, MH02:Q9C, MH03:Q9D, VT02:Q9E, MH05:Q9H, GH02:Q11B, and GH05:Q11D.

The Winsteps RMM program determines the dimensionality of a scale by using a Rasch-residual principal components analysis. When the item residuals from the RMM output were factor analysed, no significant factor loadings were present (see Table 4). This indicated that the unidimensional requirement of the SF-36 total scale was met. The raw variance explained by the SF-36 total scale over the six waves of data collection ranged from 58.5% to 62.1% and the unexplained variance in the first contrast ranged from 11.9% to 14.5%. The residual analysis completed indicated that no second dimension or factor existed. Linacre [32] suggests that a first single factor with 60% or greater of the accounted for variance is considered a reasonable unidimensional construct. “A second factor or residual factor should not indicate a substantial amount of variance if unidimensionality is tenable” [33, p. 192].

The point-measure correlation (PTMEA) ranges from +1 to -1 “with negative items suggesting improper scoring or not functioning as expected” [33, p. 192]. An inspection of the PTMEAs for the SF-36 total scale indicated that items GH01:Q1, SF01:Q6, BP01:Q7, and VT02:Q9E had consistent negative PTMEAs over the six waves of data collection. The rest of the SF-36 total scale items had PTMEAs that were positive, supporting item-level polarity. For all other items, the PTMEA correlations had acceptable values.

The functioning of the six rating scale categories was examined for the SF-36 total scale. Rating scale frequency and percent indicated that all categories were used by the participants. The category use statistics are presented in Table 5. The category logit measures ranged from -3.19 to 2.86 (see Table 5). None of the infit MNSQ scores fell outside the 0.7-1.30 range and/or a z-score that fell inside the +2 to -2 range. The results indicated that the six-level rating scale used in the SF-36 total scale fits appropriately to the predictive RMM (see Supplemental Figure 2); however, the full range of ratings were used by the participants who completed the SF-36 total scale. The probability curves for the rating scales of the six waves of data collection illustrated that each threshold estimate represented a separate point on the measure variable and each response category was the most probable category for some part of the continuum.

To investigate the possibility of item bias, differential item functioning (DIF) analysis was conducted to determine whether different groups of participants based on marital status and area of residence (urban versus regional; see Table 6) responded differently on the SF-36 total scale items, despite having the same level of the latent trait being measured [34]. Three of the SF-36 items exhibited a consistent pattern of DIF over the six waves of data collection for both marital status and area of residence, those being MH01:Q9B, MH02:Q9C, and MH05:Q9H. It should be noted that these three items also exhibited MNSQ infit scores outside the 0.7-1.30 range and/or a z-score that fell inside the +2 to -2 range.

### 3.2. SF36 Physical Health Scale Rasch Analysis for Six Waves of Data Collection

The following SF-36 physical health items were included in the initial analysis using the RMM: GH01:Q1, PF01:Q3A, PF02:Q3B, PF03:Q3C, PF04:Q3D, PF05:Q3E, PF06:Q3F, PF07:Q3G, PF08:Q3H, PF09:Q3I, PF10:Q3J, RP01:Q4A, RP02:Q4B, RP03:Q4C, RP04:Q4D, BP01:Q7, BP02:Q8, GH02:Q11A, GH03:Q11B, GH04:Q11C, and GH05:Q11D (see Table 7). When the 21 SF-36 items were calibrated using the RMM for the six waves of data collection, the items were found to have MNSQ infit statistics ranging from 0.18 to 2.66 and outfit statistics ranging from 0.19 to 2.77 (see Table 8). The mean item measure was 0.00 logits (SD = 0.99). With respect to logit measures, there was a broad range, the lowest value being –2.49 and the highest value being +1.79 (see Table 9). This resulted in an average item separation index of 60.32 and an average reliability of 1.00 over the six waves of data collection (see Table 9). The separation index for items was greater than 2.0 indicating adequate separation of the items on the construct being measured.

The SF-36 physical health scale person-item map is located in Supplemental Figure 3 and reports evidence of the hierarchical ordering of the SF-36 physical health scale items. Items which are easier are located at the bottom of the SF-36 physical health person-item map while more difficult items are located at the top of the map. The patterns of more challenging items and less difficult items on the person-item map for each of the six waves of data collection appear to be fairly consistent. It should also be noted that several of the SF-36 physical health scale items have the same level of difficulty.

The average person measure was 1.91 logits (SD = 0.39) over the six waves of data collection (see Table 9). The mean person separation was 0.93 with a mean reliability of 0.46 (see Table 9). With a mean person separation reliability of less than 2.0, this indicates inadequate separation of participants on the SF-36 physical health construct. When examining the overall RMM output of the SF-36 physical health total scale, the average person measure (1.91 logits) was higher than the average item measure (0.00 logits). The range of logit values for items was from +1.62 to -2.49 logits. The person reliability was 0.46 and item reliability was 1.00. Reliability ranges of .80 or greater are generally considered desirable [35]. This places the item reliability for the SF-36 physical health scale in the acceptable range and the person reliability correlation in the less than desired range.

The SF-36 physical health scale has a six-category rating scale which generates five thresholds. Rasch analysis reports the calibrations of the six thresholds increases monotonically from -3.86, -2.13, -.83, .10, 1.96, and 5.32 for wave one and -3.64, -2.02, -.91, .01, 2.00, and 5.24 for wave six.

Item fit to the unidimensionality requirement of the RMM was also examined. Seven out of the 21 items were found to have MNSQ infit and outfit statistics inside the 0.70 to 1.30 range and/or a z-score that fell inside the +2 to -2 range. Therefore items 1:Q1, PF01:Q3A, PF04:Q3D, PF06:Q3F, PF07:Q3G, GH02:Q11A, and GH04:Q11C met the RMM requirements (see Table 2). In other words, only 7 / 21 or 52.4% of the SF-36 physical health scale items met the RMM requirements. The following items had an Infit MNSQ statistic that was less than 0.70: PF02:Q3B, PF03:Q3C, PF05:Q3E, PF08:Q3H, PF09:Q3I, PF10:Q3J, RP01:Q4A, RP02:Q4B, RP03:Q4C, and RP04:Q4D. The following items had an Infit MNSQ statistic that was greater than 1.30: BP01:Q7, BP02:Q8, GH03:Q11B, and GH05:Q11D.

An inspection of the PTMEAs for the SF-36 physical health scale indicated that items HG01:Q1, BP01:Q7, BP02:Q8, and GH05:Q11D had consistent negative PTMEAs over the six waves of data collection. For all other items, the PTMEA correlations had acceptable values.

When the item residuals from the RMM output were factor analysed, no significant factor loadings were present (see Table 10). This indicated that the unidimensional requirement of the SF-36 physical health scale was met. The raw variance explained by the SF-36 physical health scale over the six waves of data collection ranged from 41.6% to 48.9% and the unexplained variance in the first contrast ranged from 17.4% to 22.4%. The residual analysis completed indicated that no second dimension or factor existed.

The functioning of the six rating scale categories was examined for the SF-36 physical health scale. The category logit measures ranged from -3.86 to 5.43 (see Table 11). Of the six rating scale categories, only one had infit MNSQ scores that fell outside the 0.7-1.30 range and/or a z-score that fell inside the +2 to -2 range over the six waves of data collection, this being category six. The infit MNSQ scores for this rating category ranged from 2.03 to 3.18 (see Table 11). The results indicated that the six-level rating scale used in the SF-36 physical health scale might not be the most robust to use (see Supplemental Figure 3); however, the full range of ratings were used by the participants who completed the SF-36 physical health scale. The probability curves for the rating scales of the six waves of data collection illustrated that each threshold estimate represented a separate point on the measure variable and the first five response categories were the most probable category for some part of the continuum. Rating category six was problematic.

The Rasch output logit performance scores for the participants were compared to determine if any of the SF-36 physical scale items exhibited differential item functioning (DIF), based on marital status and area of residence (urban versus regional) (see Table 12). Four of the SF-36 physical health items exhibited a consistent pattern of DIF over the six waves of data collection. Item PF03:Q3C demonstrated DIF based on marital status alone while items GH02:Q11A, GH04:Q11C, and GH05:Q11D exhibited DIF based on both marital status and area of residence (see Table 12). It should be noted that items GH02:Q11A and GH04:Q11C had infit MNSQ statistics that fell within the 0.70-1.30 range while items PF03:Q3C and GH05:Q11D also had MNSQ infit scores outside the 0.7-1.30 range and/or a z-score that fell inside the +2 to -2 range. SF-36 physical health items PF03:Q3C and GH05:Q11D appear to be particularly problematic items based on the RMM analysis findings.

### 3.3. SF36 Mental Health Scale Rasch Analysis for Six Waves of Data Collection

The following SF-36 mental health items were included in the initial analysis using the RMM: RE01:Q5A, RE02:Q5B, RE03:Q5C, SF01:Q6, VT01:Q9A, 24MH01:Q9B, MH02:Q9C, MH03:Q9D, VT02:Q9E, MH04:Q9F, VT03:Q9G, MH05:Q9H, VT04:Q9I, and SF02:Q10. When the 14 SF-36 mental health items were calibrated using the RMM for the six waves of data collection, the items were found to have MNSQ infit statistics ranging from 0.13 to 2.43 and outfit statistics ranging from 0.22 to 2.64 (see Table 13). The mean item measure was 0.00 logits (SD = 1.12). With respect to logit measures, there was a broad range, the lowest value being –3.01 and the highest value being +2.31 (see Table 14). This resulted in an average item separation index of 79.17 and an average reliability of 1.00 over the six waves (see Table 15). The separation index for items was greater than 2.0 indicating adequate separation of the items on the construct being measured.

The SF-36 mental health scale person-item map is shown in Supplemental Figure 5 and reports evidence of the hierarchical ordering of the SF-36 mental health scale items. It should also be noted that several of the SF-36 mental health scale items have the same level of difficulty. The average person measure was 0.75 logits (SD = 0.23) over the six waves of data collection (see Table 15). The mean person separation was 0.73 with a mean reliability of 0.35 (see Table 15). With a mean person separation reliability of less than 2.0, this indicates inadequate separation of participants on the SF-36 mental health construct.

When examining the overall RMM output of the SF-36 mental health scale, the average person measure (0.75 logits) was higher than the average item measure (0.00 logits). The range of logit values for items was from +2.13 to -2.08 logits. The person reliability was 0.35 and item reliability was 1.00. Reliability ranges of .80 or greater are generally considered desirable [35]. This places the item reliability for the SF-36 mental health scale in the acceptable range and the person reliability correlation in the less than desired range.

The SF-36 mental health scale has a six-category rating scale which generates five thresholds. Rasch analysis reports the calibrations of the six thresholds increases monotonically from -3.07, -1.06, -.17, .40, 1.14, and 2.54 for wave one and -2.98, -1.09, -.19, .41, 1.15, and 2.51 for wave six.

Item fit to the unidimensionality requirement of the RMM was also examined. Five out of the 14 items were found to have MNSQ infit and outfit statistics inside the 0.70 to 1.30 range and/or a z-score that fell inside the +2 to -2 range; thus, items VT01:Q9A, MH01:Q9B, MH03:Q9D, 27VT02:Q9E, MH04:Q9F, VT03:Q9G, MH05:Q9H, VT04:Q9I, and SF02:Q10 met the RMM requirements (see Table 14). In other words, only 9/14 or 64.3% of the SF-36 physical health scale items met the RMM requirements. The following items had an Infit MNSQ statistic that was less than 0.70: RE01:Q5A, RE02:Q5B, and RE03:Q5C. Item SF01:Q6 had an Infit MNSQ statistic that was greater than 1.30.

When the item residuals from the RMM output were factor analysed, no significant factor loadings were present (see Table 16). This indicated that the unidimensional requirement of the SF-36 total scale was met. The raw variance explained by the SF-36 mental health scale over the six waves of data collection ranged from 62.5% to 66.1% and the unexplained variance in the first contrast ranged from 15.1% to 16.5%.

An inspection of the PTMEAs for the SF-36 mental health scale indicated that, for all other items, the PTMEA correlations had acceptable values. All the SF-36 mental health scale items had PTMEAs that were positive, supporting item-level polarity.

The functioning of the six rating scale categories was examined for the SF-36 mental health scale. Items which are easier are located at the bottom of the SF-36 mental health person-item map while more difficult items are located at the top of the map. The patterns of more challenging items and less difficult items on the person-item map for each of the six waves of data collection appear to be fairly consistent. The category logit measures ranged from -3.86 to 2.57 (see Table 17). Of the six rating scale categories, only one had infit MNSQ scores that fell outside the 0.7-1.30 range and/or a z-score that fell inside the +2 to -2 range over the six waves of data collection, this being category one. The infit MNSQ scores for this rating category ranged from 1.38 to 1.41 (see Table 17). The results indicated that the six-level rating scale used in the SF-36 mental health scale might not be the most robust to use (see Supplemental Figure 6), however, the full range of ratings were used by the participants who completed the SF-36 mental health scale. The probability curves for the rating scales of the six waves of data collection illustrated that each threshold estimate represented a separate point on the measure variable and the latter five response categories were the most probable category for some part of the continuum. Rating category one was problematic.

The Rasch output logit performance scores for the participants were compared to determine if any of the SF-36 mental scale items exhibited differential item functioning (DIF), based on marital status and area of residence (urban versus regional) (see Table 18). Six of the SF-36 mental health items exhibited a consistent pattern of DIF over the six waves of data collection. Items SF01:Q6, MH01:Q9B, MH02:Q9C, MH03:Q9D, MH04:Q9F, and MH05:Q9H exhibited DIF based on both marital status and area of residence (see Table 18). It should be noted that items MH01:Q9B and MH03:Q9D had infit MNSQ statistics that fell outside the 0.7-1.30 range. SF-36 physical health items MH01:Q9B and MH03:Q9D appear to be particularly problematic items based on the RMM analysis findings.

## 4. Discussion

### 4.1. Is There Disordering or Dysfunction within the SF-36 Items against the Construct Being Measured?

For the SF-36 as a total measure, the rating scale categories increased monotonically, indicating that rating response scales were being used as expected and are appropriate for measurement across all waves. Previous longitudinal evaluation of the measure using CCT methods found poor test-retest reliability between two time points two weeks apart [36]. Previous research using IRT methods have been largely cross-sectional, providing little longitudinal evaluation of the measure using this method [5, 6, 10, 17]. In this sample, the pattern of more and less difficult items is consistent, indicating that item difficultly remained stable across each wave. Despite consistency across time in this sample, redundancy emerged as an issue with several total scale items displaying the same level of difficulty across all waves of data. This was seen again in both the SF-36 mental and physical health summary scores. It appears redundant items span across all uses of the measure and this suggests that item descriptors need to be more specific to avoid overlap across similar items.

Category Six of the SF-36 physical health summary scale and Category One of the SF-36 mental health scale had scores outside the acceptable range, which may indicate these rating categories are not robust for use in longitudinal studies. Disordered categories had been seen in a previous evaluation of the SF-36, with authors suggesting collapsing some category response options [5]. These findings support this issue with the SF-36. Further investigation into the category disordering in the SF-36 mental and physical health response scale is warranted and collapsing of the response option categories may improve this, as suggested in previous literature [5, 17].

When examining summary statistics for total SF-36 items, the mean person reliability fell in the unacceptable range. Inadequate person separation reliability was also seen across all waves of data, in both summary scales. The person separation index indicates the instrument used as a whole and as summary scales is not sensitive enough to separate high and low performances in the sample [29]. This presents an issue with internal consistency across all presentations of the measure. Comparatively, using classical methods, the measure was seen to discriminate between patients pre- and postoperation [37]. Results using IRT suggest that the measure is unable to discriminate between high and low performances.

While results of IRT have raised doubts of the measures internal consistency, results from classical testing methods report strong internal consistency, reflected in high Cronbach's alpha scores. When validating the measure in patients with endometriosis, Cronbach's alpha for the total scale was above acceptable cut-offs [38]. Internal consistency scores have also been seen to be above .9 for the full scale and above .7 for each subscale [39]. In addition to internal consistency, the measure displayed acceptable content validity, correlating strongly with similar measures [38]. IRT assesses instrument reliability at item level, rather than instrument-level as well as considering considers the importance of participant responses.

The contrast between results from IRT and CTT could be due to the further focus at item level that is characteristics to IRT. It is possible that overlapping items identified in the person-item map are contributing to lack of sensitivity in the scale. Addition of more items or altering current items to improve sensitivity may improve the person reliability. Further investigation into the similarity and specificity of these items is warranted, to ensure items capture the full variable being measured.

### 4.2. Do the SF-36 Items Have a Consistent Hierarchy and Good Distribution across All Waves?

Several items on the total scale and both summary scales were found to have Infit statistics outside of the acceptable range. Many of the items remained problematic regardless of investigated as whole measure or by summary scale. The number of misfitting items was slightly lower when used in summary scales; however this can be due to the less items included in the summary scale analysis. These underfitting items create concerns about degradation of the model and the validity of the measure as a measure of health related quality of life [15]. Further investigation into such items is required to determine the reason for underfit. While overfit items do not degrade the model, they can result in misinterpretation of the model as working better than expected and also warrant further investigation [15].

### 4.3. Does the SF-36 Measure One or More Constructs?

The measure proved to be unidimensional across total scale and summary score analyses, indicating responses to each scale are likely to be determined by a single trait. As a total scale, the first single factor accounted for close to 60% across all six waves and the factor was considered unidimensional [32]. Residual analysis also indicated no second dimension or factor existed, further confirming unidimensioanlity of the total scale [33]. Analysis of all eight subscales revealed each scale measured a single latent trait [6]. Principal components analysis of the physical and mental health summary scores has confirmed the presence of a two-factor model, further corroborated by the results of the current study support the mental and physical health scales [12].

Results suggest the responses to measure are determined by a single factor. While the responses may be determined by a single factor, previously identified misfitting and overlapping items may degrade the model and validity, suggesting that it may not be health-related quality of life that is determining response to these items. Further research should aim to correct misfitting items and reassess unidimensionality.

### 4.4. Were All Items in the SF-36 Instrument Used by All Groups in the Same Way?

It appears that marital status and area of residence influence responses to both total and summary scale items. Differential item functioning has identified in the SF-36 previously, with health issues such as hypertension, respiratory issues, and diabetes influencing responses on five items in the measure [10]. Previously, the presence of DIF has been considered negligible, as it was only present for a small number of items [10]. As the SF-36 is a health-related quality of life measure, it is plausible that marital status or area of residence would have an impact in this domain as these factors can influence healthcare use and quality of life. However, the presence of DIF limits the ability of scores to be comparable across different populations.

While several items on each summary scale and total scale exhibited DIF, only item 24:Q9B demonstrated DIF across analysis of total scale and items in the summary scales. This particular item also demonstrated Infit statistics outside the acceptable range, proving to be particularly problematic in every presentation of the measure. Several other items demonstrated DIF and misfit. Given that the number of items exhibiting DIF and misfit across all presentations of the measure, further investigation is needed into these specific items.

### 4.5. Limitations and Future Research

While the current study revealed differences between IRT and CTT evaluations of the SF-36, it did not compare each method in the same sample. Future research may perform both methods using the same sample, in order to explain the differences between methods and advantages of applying different frameworks when developing and evaluating measures. It may also be beneficial to compare methods longitudinally. A further limitation is the rate of attrition in the sample. While attrition is to be expected in a longitudinal study, results between waves should be interpreted in light of this.

The results suggest the SF-36 is not as sound as previously suggested. It can be delivered as eight subscales and future research may apply the RMM to each subscale to evaluate the efficacy of the measure in this form. Based on the RMM findings in the current study, future research should further evaluate this measure using IRT methods. Results suggest multiple items needed to be reassessed to avoid degrading the model and improve performance of the SF-36 as a reliable measure of health-related quality of life.

## 5. Conclusions

Previous evaluations of the SF-36 have relied on cross-sectional data; however, the findings of the current study demonstrate the longitudinal efficacy of the measure. While using of the measure remained consistent across time for both the whole measure and summary scales, several issues were identified. Previous studies evaluating the SF-36 using CCT methods describe the measure as reliable and valid. However, evaluating the measure by application of the RMM indicated issues with internal consistency, generalisability, and sensitivity when the measure was evaluated as a whole and as both physical and mental health summary scales.

## Figures and Tables

**Table 1:** SF-36 total scale Rasch analysis item statistics for six waves of data collection.

	**Wave 1**			**Wave 2 **			**Wave 3 **			**Wave 4 **			**Wave 5 **			**Wave 6 **		
**SF36 ITEM**	**LOGIT MEASURE**	**MODEL S.E**	**PTMEA CORR**	**LOGIT MEASURE**	**MODEL S.E.**	**PTMEA CORR**	**LOGIT MEASURE**	**MODEL S.E.**	**PTMEA CORR**	**LOGIT MEASURE**	**MODEL S.E.**	**PTMEA CORR**	**LOGIT MEASURE**	**MODEL S.E.**	**PTMEA CORR**	**LOGIT MEASURE**	**MODEL S.E.**	**PTMEA CORR**
**1: Q1 **	-0.36	0.01	-0.16	-0.37	0.01	-0.14	-0.37	0.01	-0.09	-0.52	0.01	-0.06	-0.60	0.01	-0.05	-0.68	0.01	0.01
**2: Q2 **	-0.39	0.01	0.04	-0.44	0.01	0.03	-0.51	0.01	0.03	-0.56	0.01	0.08	-0.66	0.01	0.06	-0.73	0.01	0.11
**3: Q3A **	1.94	0.02	0.24	1.96	0.02	0.26	2.05	0.02	0.29	2.08	0.02	0.30	2.09	0.03	0.29	2.31	0.04	0.27
**4: Q3B **	0.36	0.01	0.46	0.39	0.01	0.44	0.53	0.01	0.44	0.67	0.01	0.45	0.77	0.02	0.45	0.95	0.02	0.41
**5: Q3C **	0.30	0.01	0.47	0.32	0.01	0.46	0.34	0.01	0.44	0.44	0.01	0.46	0.49	0.02	0.44	0.57	0.02	0.41
**6: Q3D **	0.82	0.01	0.45	0.84	0.01	0.44	0.93	0.01	0.46	1.00	0.02	0.47	1.11	0.02	0.46	1.25	0.02	0.44
**7: Q3E **	0.13	0.01	0.51	0.15	0.01	0.50	0.24	0.01	0.50	0.28	0.01	0.49	0.36	0.02	0.49	0.46	0.02	0.47
**8: Q3F **	0.54	0.01	0.44	0.63	0.01	0.41	0.72	0.01	0.41	0.73	0.02	0.43	0.73	0.02	0.43	0.81	0.02	0.40
**9: Q3G **	0.44	0.01	0.49	0.53	0.01	0.46	0.73	0.01	0.46	0.87	0.02	0.47	1.05	0.02	0.44	1.24	0.02	0.44
**10: Q3H **	0.05	0.01	0.52	0.10	0.01	0.49	0.23	0.01	0.49	0.36	0.01	0.50	0.48	0.02	0.48	0.65	0.02	0.48
**11: Q3I **	-0.14	0.01	0.48	-0.13	0.01	0.45	-0.07	0.01	0.46	-0.02	0.01	0.44	0.05	0.01	0.44	0.11	0.02	0.45
**12: Q3J **	-0.28	0.01	0.36	-0.31	0.01	0.35	-0.29	0.01	0.32	-0.24	0.01	0.32	-0.23	0.01	0.32	-0.23	0.02	0.32
**13: Q4A **	1.26	0.01	0.35	1.31	0.01	0.33	1.41	0.02	0.29	1.41	0.02	0.28	1.46	0.02	0.26	1.47	0.03	0.27
**14: Q4B **	1.63	0.01	0.35	1.74	0.02	0.32	1.87	0.02	0.29	1.89	0.02	0.28	1.92	0.03	0.27	1.94	0.03	0.26
**15: Q4C **	1.47	0.01	0.36	1.53	0.02	0.36	1.60	0.02	0.31	1.69	0.02	0.30	1.74	0.02	0.28	1.78	0.03	0.24
**16: Q4D **	1.50	0.01	0.36	1.58	0.02	0.35	1.67	0.02	0.31	1.73	0.02	0.30	1.77	0.02	0.28	1.80	0.03	0.28
**17: Q5A **	1.02	0.01	0.37	1.01	0.01	0.35	1.00	0.01	0.31	0.96	0.02	0.30	0.92	0.02	0.30	0.89	0.02	0.27
**18: Q5B **	1.22	0.01	0.36	1.22	0.01	0.35	1.23	0.02	0.31	1.21	0.02	0.30	1.18	0.02	0.29	1.15	0.02	0.27
**19: Q5C **	1.05	0.01	0.35	1.03	0.01	0.33	1.01	0.01	0.31	0.99	0.02	0.29	0.95	0.02	0.29	0.91	0.02	0.26
**20: Q6 **	1.17	0.01	-0.22	1.35	0.01	-0.20	0.93	0.01	-0.16	0.69	0.01	-0.16	0.48	0.02	-0.12	0.37	0.02	-0.08
**21: Q7 **	-0.28	0.01	-0.06	-0.26	0.01	-0.04	-0.40	0.01	0.01	-0.50	0.01	0.01	-0.57	0.01	0.03	-0.67	0.01	0.07
**22: Q8 **	0.68	0.01	-0.18	0.73	0.01	-0.14	0.44	0.01	-0.11	0.26	0.01	-0.09	0.12	0.01	-0.04	-0.01	0.02	-0.02
**23: Q9A **	-0.59	0.01	-0.05	-0.67	0.01	-0.06	-0.79	0.01	0.00	-0.82	0.01	-0.02	-0.93	0.01	0.01	-1.06	0.01	0.05
**24: Q9B **	-2.04	0.01	0.39	-2.30	0.01	0.36	-2.39	0.01	0.33	-2.40	0.01	0.31	-2.38	0.02	0.31	-2.42	0.02	0.27
**25: Q9C **	-2.64	0.01	0.40	-2.92	0.02	0.35	-2.98	0.02	0.34	-3.01	0.02	0.30	-2.86	0.02	0.31	-2.89	0.02	0.27
**26: Q9D **	-0.30	0.01	0.06	-0.20	0.01	0.01	-0.28	0.01	0.09	-0.29	0.01	0.07	-0.30	0.01	0.08	-0.37	0.01	0.12
**27: Q9E **	-0.77	0.01	-0.05	-0.89	0.01	-0.09	-1.00	0.01	-0.04	-1.06	0.01	-0.05	-1.17	0.01	-0.02	-1.35	0.01	0.00
**28: Q9F **	-2.01	0.01	0.40	-2.15	0.01	0.34	-2.15	0.01	0.35	-2.16	0.01	0.34	-2.13	0.02	0.33	-2.13	0.02	0.31
**29: Q9G **	-1.63	0.01	0.44	-1.70	0.01	0.41	-1.67	0.01	0.40	-1.60	0.01	0.40	-1.56	0.01	0.40	-1.57	0.01	0.37
**30: Q9H **	0.24	0.01	0.14	0.33	0.01	0.09	0.26	0.01	0.13	0.23	0.01	0.14	0.14	0.01	0.12	0.08	0.02	0.15
**31: Q9I **	-1.23	0.01	0.39	-1.22	0.01	0.37	-1.15	0.01	0.34	-1.07	0.01	0.36	-1.03	0.01	0.34	-1.04	0.01	0.31
**32: Q10 **	-1.34	0.01	0.35	-1.39	0.01	0.31	-1.30	0.01	0.28	-1.23	0.01	0.26	-1.20	0.01	0.24	-1.18	0.01	0.24
**33: Q11A**	-1.39	0.01	0.33	-1.48	0.01	0.31	-1.49	0.01	0.28	-1.50	0.01	0.25	-1.49	0.01	0.26	-1.52	0.01	0.20
**34: Q11B**	0.28	0.01	0.03	0.43	0.01	-0.01	0.32	0.01	0.08	0.25	0.01	0.07	0.10	0.01	0.10	-0.01	0.02	0.14
**35: Q11C**	-0.68	0.01	0.29	-0.75	0.01	0.27	-0.65	0.01	0.29	-0.59	0.01	0.25	-0.50	0.01	0.25	-0.48	0.01	0.24
**36: Q11D**	-0.02	0.01	-0.06	0.01	0.01	-0.09	-0.03	0.01	0.00	-0.17	0.01	-0.01	-0.31	0.01	0.04	-0.40	0.01	0.09

*Note*. MODEL S.E. = model standard error; PTMEA CORR = point measure correlation.

**Table 2:** SF-36 total scale Rasch analysis Infit and Outfit statistics for six waves of data collection.

	**Wave 1**				**Wave 2**				**Wave 3**			
**SF36 ITEM**	**INFIT**		**Outfit**		**Infit**		**Outfit**		**Infit**		**Outfit**	
	**MNSQ**	**ZSTD**	**MNSQ**	**ZSTD**	**MNSQ**	**ZSTD**	**MNSQ**	**ZSTD**	**MNSQ**	**ZSTD**	**MNSQ**	**ZSTD**

**1: Q1 **	0.92	-6.7	0.96	-2.9	0.86	-9.9	0.90	-7.3	0.83	-9.9	0.86	-9.8
**2: Q2 **	0.51	-9.9	0.55	-9.9	0.53	-9.9	0.57	-9.9	0.54	-9.9	0.56	-9.9
**3: Q3A **	1.03	2.1	1.02	1.4	1.12	7.0	1.09	5.5	1.06	3.0	1.02	1.2
**4: Q3B **	0.59	-9.9	0.61	-9.9	0.64	-9.9	0.65	-9.9	0.72	-9.9	0.73	-9.9
**5: Q3C **	0.54	-9.9	0.56	-9.9	0.56	-9.9	0.57	-9.9	0.59	-9.9	0.60	-9.9
**6: Q3D **	0.82	-9.9	0.82	-9.9	0.84	-9.9	0.84	-9.9	0.86	-8.6	0.86	-8.7
**7: Q3E **	0.46	-9.9	0.48	-9.9	0.48	-9.9	0.50	-9.9	0.54	-9.9	0.56	-9.9
**8: Q3F **	0.66	-9.9	0.67	-9.9	0.72	-9.9	0.72	-9.9	0.75	-9.9	0.75	-9.9
**9: Q3G **	0.76	-9.9	0.78	-9.9	0.83	-9.9	0.85	-9.9	0.94	-3.7	0.94	-3.4
**10: Q3H **	0.46	-9.9	0.50	-9.9	0.53	-9.9	0.56	-9.9	0.65	-9.9	0.68	-9.9
**11: Q3I **	0.27	-9.9	0.30	-9.9	0.29	-9.9	0.31	-9.9	0.37	-9.9	0.39	-9.9
**12: Q3J **	0.17	-9.9	0.19	-9.9	0.13	-9.9	0.15	-9.9	0.17	-9.9	0.18	-9.9
**13: Q4A **	0.40	-9.9	0.41	-9.9	0.41	-9.9	0.42	-9.9	0.49	-9.9	0.50	-9.9
**14: Q4B **	0.57	-9.9	0.58	-9.9	0.61	-9.9	0.61	-9.9	0.66	-9.9	0.66	-9.9
**15: Q4C **	0.50	-9.9	0.51	-9.9	0.51	-9.9	0.52	-9.9	0.57	-9.9	0.58	-9.9
**16: Q4D **	0.51	-9.9	0.53	-9.9	0.54	-9.9	0.55	-9.9	0.59	-9.9	0.60	-9.9
**17: Q5A **	0.25	-9.9	0.27	-9.9	0.22	-9.9	0.23	-9.9	0.25	-9.9	0.26	-9.9
**18: Q5B **	0.37	-9.9	0.39	-9.9	0.36	-9.9	0.37	-9.9	0.39	-9.9	0.41	-9.9
**19: Q5C **	0.27	-9.9	0.29	-9.9	0.24	-9.9	0.26	-9.9	0.26	-9.9	0.28	-9.9
**20: Q6 **	*2.43*	9.9	*2.59*	9.9	*2.48*	9.9	*2.64*	9.9	*2.36*	9.9	*2.48*	9.9
**21: Q7 **	*1.84*	9.9	*1.90*	9.9	*1.96*	9.9	*2.01*	9.9	*1.70*	9.9	*1.73*	9.9
**22: Q8 **	*2.09*	9.9	*2.17*	9.9	*2.14*	9.9	*2.21*	9.9	*1.93*	9.9	*1.99*	9.9
**23: Q9A **	*1.48*	9.9	*1.52*	9.9	*1.48*	9.9	*1.52*	9.9	*1.44*	9.9	*1.46*	9.9
**24: Q9B **	*1.47*	9.9	*1.40*	9.9	*1.64*	9.9	*1.55*	9.9	*1.64*	9.9	*1.55*	9.9
**25: Q9C **	*1.67*	9.9	*1.53*	9.9	*1.82*	9.9	*1.66*	9.9	*1.74*	9.9	*1.57*	9.9
**26: Q9D **	*1.69*	9.9	*1.73*	9.9	*1.60*	9.9	*1.64*	9.9	*1.50*	9.9	*1.52*	9.9
**27: Q9E **	*1.55*	9.9	*1.58*	9.9	*1.55*	9.9	*1.58*	9.9	*1.49*	9.9	*1.50*	9.9
**28: Q9F **	1.07	5.1	1.03	2.4	1.19	9.9	1.15	9.0	1.13	6.9	1.09	4.9
**29: Q9G **	1.02	2.0	1.00	0.2	1.04	3.0	1.02	1.4	1.02	1.1	1.00	-0.1
**30: Q9H **	*1.63*	9.9	*1.62*	9.9	*1.49*	9.9	*1.50*	9.9	*1.35*	9.9	*1.36*	9.9
**31: Q9I **	0.84	-9.9	0.84	-9.9	0.88	-9.9	0.88	-9.9	0.84	-9.9	0.84	-9.9
**32: Q10 **	0.79	-9.9	0.79	-9.9	0.84	-9.9	0.83	-9.9	0.92	-6.6	0.92	-6.6
**33: Q11A**	0.77	-9.9	0.76	-9.9	0.64	-9.9	0.63	-9.9	0.66	-9.9	0.65	-9.9
**34: Q11B**	*1.88*	9.9	*1.90*	9.9	*1.77*	9.9	*1.80*	9.9	*1.62*	9.9	*1.62*	9.9
**35: Q11C**	1.04	3.3	1.05	4.1	0.95	-4.5	0.95	-3.8	0.99	-0.9	0.99	-0.5
**36: Q11D**	*1.78*	9.9	*1.83*	9.9	*1.81*	9.9	*1.86*	9.9	*1.68*	9.9	*1.70*	9.9

	**Wave 4**				**Wave 5**				**Wave 6**			
**SF36 ITEM**	**Infit**		**Outfit**		**Infit**		**Outfit**		**Infit**		**Outfit**	
	**MNSQ**	**ZSTD**	**MNSQ**	**ZSTD**	**MNSQ**	**ZSTD**	**MNSQ**	**ZSTD**	**MNSQ**	**ZSTD**	**MNSQ**	**ZSTD**

**1: Q1 **	0.72	-9.9	0.73	-9.9	0.69	-9.9	0.70	-9.9	0.65	-9.9	0.65	-9.9
**2: Q2 **	0.50	-9.9	0.52	-9.9	0.49	-9.9	0.50	-9.9	0.46	-9.9	0.47	-9.9
**3: Q3A **	1.11	4.4	1.06	2.3	1.18	6.0	1.11	3.9	1.26	6.4	1.17	4.3
**4: Q3B **	0.78	-9.9	0.78	-9.9	0.81	-9.1	0.81	-9.4	0.91	-3.6	0.90	-4.1
**5: Q3C **	0.62	-9.9	0.64	-9.9	0.65	-9.9	0.66	-9.9	0.72	-9.9	0.72	-9.9
**6: Q3D **	0.87	-7.1	0.86	-7.7	0.92	-3.6	0.90	-4.6	0.97	-0.9	0.93	-2.4
**7: Q3E **	0.56	-9.9	0.57	-9.9	0.61	-9.9	0.63	-9.9	0.70	-9.9	0.70	-9.9
**8: Q3F **	0.73	-9.9	0.73	-9.9	0.71	-9.9	0.72	-9.9	0.76	-9.9	0.76	-9.9
**9: Q3G **	0.98	-1.0	0.97	-1.3	1.03	1.5	1.01	0.6	1.09	3.3	1.04	1.6
**10: Q3H **	0.74	-9.9	0.76	-9.9	0.83	-8.0	0.85	-7.4	0.94	-2.4	0.93	-2.6
**11: Q3I **	0.41	-9.9	0.43	-9.9	0.48	-9.9	0.51	-9.9	0.55	-9.9	0.57	-9.9
**12: Q3J **	0.24	-9.9	0.26	-9.9	0.29	-9.9	0.31	-9.9	0.33	-9.9	0.34	-9.9
**13: Q4A **	0.52	-9.9	0.54	-9.9	0.58	-9.9	0.60	-9.9	0.60	-9.9	0.61	-9.9
**14: Q4B **	0.69	-9.9	0.69	-9.9	0.73	-9.9	0.72	-9.9	0.74	-8.7	0.74	-8.9
**15: Q4C **	0.62	-9.9	0.63	-9.9	0.67	-9.9	0.68	-9.9	0.71	-9.9	0.71	-9.9
**16: Q4D **	0.64	-9.9	0.64	-9.9	0.68	-9.9	0.68	-9.9	0.71	-9.9	0.71	-9.9
**17: Q5A **	0.27	-9.9	0.29	-9.9	0.30	-9.9	0.32	-9.9	0.32	-9.9	0.34	-9.9
**18: Q5B **	0.42	-9.9	0.43	-9.9	0.45	-9.9	0.47	-9.9	0.47	-9.9	0.49	-9.9
**19: Q5C **	0.29	-9.9	0.31	-9.9	0.32	-9.9	0.34	-9.9	0.33	-9.9	0.35	-9.9
**20: Q6 **	*2.17*	9.9	*2.27*	9.9	*2.06*	9.9	*2.14*	9.9	*2.00*	9.9	*2.06*	9.9
**21: Q7 **	*1.61*	9.9	*1.63*	9.9	*1.52*	9.9	*1.53*	9.9	*1.40*	9.9	*1.41*	9.9
**22: Q8 **	*1.75*	9.9	*1.80*	9.9	*1.65*	9.9	*1.68*	9.9	*1.56*	9.9	*1.59*	9.9
**23: Q9A **	*1.40*	9.9	*1.41*	9.9	*1.36*	9.9	*1.36*	9.9	*1.34*	9.9	*1.35*	9.9
**24: Q9B **	*1.62*	9.9	*1.53*	9.9	*1.61*	9.9	*1.53*	9.9	*1.58*	9.9	*1.51*	9.9
**25: Q9C **	*1.73*	9.9	*1.60*	9.9	*1.75*	9.9	*1.62*	9.9	*1.65*	9.9	*1.57*	9.9
**26: Q9D **	*1.51*	9.9	*1.53*	9.9	*1.47*	9.9	*1.49*	9.9	*1.41*	9.9	*1.42*	9.9
**27: Q9E **	*1.51*	9.9	*1.52*	9.9	*1.44*	9.9	*1.45*	9.9	*1.46*	9.9	*1.47*	9.9
**28: Q9F **	1.15	7.5	1.11	5.5	1.16	7.0	1.12	5.3	1.12	4.6	1.09	3.6
**29: Q9G **	1.02	1.6	1.01	0.5	1.03	1.6	1.01	0.8	1.06	2.7	1.04	2.1
**30: Q9H **	*1.36*	9.9	*1.36*	9.9	*1.35*	9.9	*1.37*	9.9	1.29	9.9	1.29	9.9
**31: Q9I **	0.89	-8.2	0.89	-7.9	0.92	-5.2	0.92	-4.9	0.91	-5.1	0.91	-4.8
**32: Q10 **	0.93	-5.1	0.94	-4.7	1.00	-0.2	1.00	0.0	1.06	3.0	1.06	3.1
**33: Q11A**	0.67	-9.9	0.66	-9.9	0.67	-9.9	0.66	-9.9	0.70	-9.9	0.69	-9.9
**34: Q11B**	*1.58*	9.9	*1.59*	9.9	*1.44*	9.9	*1.45*	9.9	*1.38*	9.9	*1.38*	9.9
**35: Q11C**	1.01	0.5	1.01	0.9	0.99	-0.6	1.00	-0.2	0.98	-1.0	0.98	-0.9
**36: Q11D**	*1.61*	9.9	*1.64*	9.9	*1.51*	9.9	*1.53*	9.9	*1.43*	9.9	*1.43*	9.9

*Notes*. MNSQ = mean square residual fit statistic; ZSTD: standardized mean square residual fit statistic; Z-STD ≤-2.0 or ≥2.0; values in italic for Infit or Outfit MnSq > 1.34; values underlined for Infit or Outfit MnSq < 0.64.

**Table 3:** SF-36 total scale Rasch analysis summary Item and Person Infit and Outfit statistics for six waves of data collection.

		**Wave 1**	**Wave 2 **	**Wave 3**	**Wave 4 **	**Wave 5 **	**Wave 6 **
**Persons**	**Mean**	-.69	-.68	-.72	-.75	-.80	-.85
	**S.D.**	.24	.22	.24	.23	.23	.24
	**MAX**	.82	.29	.64	.67	.03	.15
	**MIN**	-4.33	-2.70	-2.60	-2.59	-2.54	-2.76
	**Infit-MNSQ**	1.03	1.02	1.03	1.04	1.04	1.03
	**Infit-ZSTD**	-.30	-.40	-.30	-.20	-.20	-.10
	**Outfit-MNSQ**	1.01	1.01	1.00	1.00	.99	.99
	**Outfit-ZSTD**	-.40	-.40	-.30	-.30	-.30	-.20
****	**Person separation**	.81^c^	.60^c^	.72^c^	.71^c^	.75^c^	.78^c^
	**Person reliability**	.40^a^	.26^a^	.34^a^	.33^a^	.36^a^	.38^a^

**Items**	**Mean**	.00	.00	.00	.00	.00	.00
	**S.D.**	1.11	1.19	1.20	1.21	1.22	1.26
	**MAX**	1.94	1.96	2.05	2.08	2.09	2.31
	**MIN**	-2.64	-2.92	-2.98	-3.01	-2.86	-2.89
	**Infit-MNSQ**	.98	.99	.98	.98	.98	.99
	**Infit-ZSTD**	-2.30	-2.30	-2.20	-1.90	-1.40	-.90
	**Outfit-MNSQ**	.99	1.00	.98	.98	.98	.98
	**Outfit-ZSTD**	-2.30	-2.30	-2.30	-2.00	-1.50	-1.10
	**Item separation**	93.40	89.72	82.81	76.45	67.43	58.09
****	**Item reliability**	1.00	1.00	1.00	1.00	1.00	1.00

*Notes*. ^a^Person or item reliability <0.8; ^b^Item separation <3.0; ^c^Person separation <2.0; values in italic for Infit or Outfit MnSq > 1.34; values underlined for Infit or Outfit MnSq < 0.64.

**Table 4:** SF-36 total scale Rasch analysis of standardised residual variance in Eigenvalue units for six waves of data collection.

	**Wave 1**			**Wave 2**			**Wave 3**		
	**Eigenvalue**	**Observed**	**Expected**	**Eigenvalue**	**Observed**	**Expected**	**Eigenvalue**	**Observed**	**Expected**
**Total raw variance in observations**	86.71	100.00%	100.00%	92.22	100.00%	100.00%	92.42	100.00%	100.00%
**Raw variance explained by measures**	50.71	58.50%	58.70%	56.22	61.00%	61.10%	56.42	61.00%	61.30%
**Raw variance explained by persons**	3.47	4.00%	4.00%	1.93	2.10%	2.10%	2.04	2.20%	2.20%
**Raw Variance explained by items**	47.24	54.50%	54.70%	54.30	58.90%	59.00%	54.38	58.80%	59.10%
**Raw unexplained variance (total)**	36.00	41.50%	41.30%	36.00	39.00%	38.90%	36.00	39.00%	38.70%
**Unexplained variance in 1st contrast**	12.60	14.50%	35.00%	12.57	13.60%	34.90%	12.26	13.30%	34.10%
**Unexplained variance in 2nd contrast**	3.02	3.50%	8.40%	3.05	3.30%	8.50%	3.03	3.30%	8.40%
**Unexplained variance in 3rd contrast**	1.89	2.20%	5.20%	1.78	1.90%	4.90%	1.84	2.00%	5.10%
**Unexplained variance in 4th contrast**	1.59	1.80%	4.40%	1.54	1.70%	4.30%	1.50	1.60%	4.20%
**Unexplained variance in 5th contrast**	1.24	1.40%	3.40%	1.27	1.40%	3.50%	1.26	1.40%	3.50%

	**Wave 4**			**Wave 5**			**Wave 6**		
	**Eigenvalue**	**Observed**	**Expected**	**Eigenvalue**	**Observed**	**Expected**	**Eigenvalue**	**Observed**	**Expected**

**Total raw variance in observations**	92.10	100.00%	100.00%	91.96	100.00%	100.00%	94.92	100.00%	100.00%
**Raw variance explained by measures**	56.10	60.90%	61.50%	55.96	60.90%	61.70%	58.92	62.10%	63.00%
**Raw variance explained by persons**	3.59	3.90%	3.90%	4.05	4.40%	4.50%	4.57	4.80%	4.90%
**Raw Variance explained by items**	52.51	57.00%	57.60%	51.91	56.40%	57.20%	54.35	57.30%	58.10%
**Raw unexplained variance (total)**	36.00	39.10%	38.50%	36.00	39.10%	38.30%	36.00	37.90%	37.00%
**Unexplained variance in 1st contrast**	12.41	13.50%	34.50%	12.08	13.10%	33.60%	11.33	11.90%	31.50%
**Unexplained variance in 2nd contrast**	3.06	3.30%	8.50%	3.20	3.50%	8.90%	3.22	3.40%	8.90%
**Unexplained variance in 3rd contrast**	1.88	2.00%	5.20%	1.95	2.10%	5.40%	2.17	2.30%	6.00%
**Unexplained variance in 4th contrast**	1.50	1.60%	4.20%	1.53	1.70%	4.30%	1.55	1.60%	4.30%
**Unexplained variance in 5th contrast**	1.27	1.40%	3.50%	1.25	1.40%	3.50%	1.30	1.40%	3.60%

*Notes.* ^a^ > 60% unexplained variance in the Rasch factor; ^b^ Eigenvalue in the first contrast <3.0; ^c^ < 10% unexplained variance in the first contrast.

**Table 5:** SF-36 total scale Rasch analysis of summary of category structure for six waves of data collection

	**Wave 1**						**Wave 2**					
**Cat. Label**	**N**	**%**	**Average Measures**	**Infit MnSq**	**Outfit MnSq**	**Andrich Threshold**	**N**	**%**	**Average Measures**	**Infit MnSq**	**Outfit MnSq**	**Andrich Threshold**
1	84119	19	(-3.15)	1.30	1.17	NONE	72530	19	(-3.19)	1.28	1.17	NONE
2	133566	30	-1.36	.99	1.01	-1.88	114964	31	-1.40	1.02	1.06	-1.93
3	96735	22	-.25	.66	.61	-.54	82817	22	-.26	.67	.63	-.57
4	40204	9	.48	.98	1.04	.61	34325	9	.50	.97	1.03	.61
5	44040	10	1.31	1.06	1.17	.28^b^	37154	10	1.34	1.07	1.20	.34^b^
6	25211	6	(2.82)	.93	1.01	1.53	23593	6	(2.86)	.92	.99	1.56

	**Wave 3**						**Wave 4**					
**Cat. Label**	**N**	**%**	**Average Measures**	**Infit MnSq**	**Outfit MnSq**	**Andrich Threshold**	**N**	**%**	**Average Measures**	**Infit MnSq**	**Outfit MnSq**	**Andrich Threshold**

1	61757	20	(-3.14)	1.25	1.15	NONE	55809	22	(-3.06)	1.24	1.15	NONE
2	88701	29	-1.39	1.04	1.08	-1.87	72286	28	-1.35	1.05	1.10	-1.79
3	63285	20	-.28	.73	.67	-.57	50125	19	-.30	.78	.71	-.54
4	29486	9	.48	.94	.96	.48	25807	10	.45	.90	.88	.38
5	28991	9	1.34	1.09	1.16	.41^b^	24560	10	1.32	1.10	1.13	.41
6	18470	6	(2.86)	.87	.93	1.55	15297	6	(2.85)	.86	.91	1.54

	**Wave 5**						**Wave 6**					
**Cat. Label**	**N**	**%**	**Average Measures**	**Infit MnSq**	**Outfit MnSq**	**Andrich Threshold**	**N**	**%**	**Average Measures**	**Infit MnSq**	**Outfit MnSq**	**Andrich Threshold**

1	47041	24	(-3.00)	1.26	1.16	NONE	36377	25	(-2.96)	1.23	1.14	NONE
2	54905	27	-1.31	1.06	1.09	-1.72	37987	26	-1.30	1.06	1.07	-1.67
3	36216	18	-.30	.83	.76	-.49	24952	17	-.31	.88	.83	-.49
4	21172	11	.43	.87	.81	.27	15554	11	.42	.87	.79	.23
5	18847	9	1.29	1.13	1.15	.46	13560	9	1.29	1.15	1.15	.50
6	11583	6	(2.80)	.83	.88	1.48	8495	6	(2.78)	.83	.88	1.44

*Notes.* ^a^Andrich threshold category increase of >5; ^b^Andrich threshold category decrease where an increase is expected; values in italic for Infit or Outfit MnSq > 1.34; values underlined for Infit or Outfit MnSq < 0.64.

**Table 6:** Differential Item Functioning (DIF) for SF-36 total scale Rasch analysis for six waves of data collection based on marital status and area of residence.

	WAVE 1				WAVE 2				Wave 3			
	Marital status		Urban and Regional		Marital status		Urban and Regional		Marital status		Urban and Regional	
ITEM	SUMMARY DIF	PROB.	DIF CONTRAST	Mantel- Haenszel Probability	SUMMARY DIF	PROB.	DIF CONTRAST	Mantel- Haenszel Probability	SUMMARY DIF	PROB.	DIF CONTRAST	Mantel- Haenszel Probability
No.	CHI-SQUARE (DIF=2)				CHI-SQUARE (DIF=2)				CHI-SQUARE (DIF=2)			

1	8.68	.013^*∗∗*^	0.00	.924	1.78	.407	0.19	.452	0.25	.882	0.00	.861
2	3.17	.202	0.00	.295	0.55	.760	0.00	.791	0.14	.936	0.00	.134
3	4.95	.083	0.00	.811	0.21	.903	0.20	.326	5.47	.064	0.00	.907
4	0.87	.647	0.00	.492	0.44	.804	0.00	.010^*∗*^	0.34	.847	0.00	.438
5	0.89	.640	0.05	.001^*∗∗∗*^	0.09	.959	-0.14	.983	0.40	.818	0.02	.002^*∗∗*^
6	0.47	.792	0.00	.142	4.67	.095	0.00	.288	0.57	.750	0.00	.619
7	0.06	.971	0.00	.687	2.94	.227	-0.01	.800	1.39	.496	-0.02	.054
8	0.32	.855	0.00	.362	0.27	.875	0.02	.033^*∗*^	0.06	.974	0.00	.243
9	13.66	.001^*∗∗∗*^	-0.06	.003^*∗∗*^	2.06	.354	-0.14	.603	0.70	.704	-0.06	.072
10	11.27	.004^*∗∗*^	0.00	.030^*∗*^	7.04	.029^*∗*^	0.06	.001^*∗∗∗*^	0.00	1.000	-0.06	.006^*∗∗*^
11	3.41	.179	0.00	.071	6.25	.043^*∗*^	0.04	.503	0.00	1.000	0.00	.473
12	0.16	.926	0.00	.906	0.10	.952	0.00	.981	0.69	.706	0.00	.722
13	2.93	.227	0.00	.845	0.09	.959	-0.19	.474	0.04	.982	0.00	.159
14	0.96	.618	0.00	.327	3.10	.210	0.00	.822	6.13	.046^*∗*^	-0.05	.126
15	2.37	.303	0.00	.366	0.06	.970	0.07	.660	0.38	.828	0.00	.815
16	0.00	1.000	0.00	.591	0.05	.976	0.00	.358	4.14	.124	0.00	.317
17	1.80	.404	0.00	.581	0.10	.952	-0.02	.956	0.03	.987	0.00	.475
18	3.78	.149	0.00	.704	0.52	.770	-0.02	.238	0.62	.731	0.00	.571
19	1.54	.460	0.00	.892	0.23	.893	-0.10	.836	0.06	.971	0.00	.882
20	55.71	.001^*∗∗∗*^	-0.07	.036	7.62	.022^*∗*^	0.00	.526	0.06	.970	0.00	.088
21	3.24	.195	-0.06	.011^*∗*^	1.17	.554	0.15	.087	0.00	1.000	0.00	.784
22	33.92	.001^*∗∗∗*^	0.00	.239	4.09	.127	0.00	.661	1.52	.465	0.03	.649
23	7.12	.028^*∗*^	0.00	.100	2.90	.231	-0.06	.436	0.18	.916	0.00	.498
24	23.59	.001^*∗∗∗*^	0.11	.001^*∗∗∗*^	0.12	.942	0.00	.993	13.40	.001^*∗∗∗*^	0.00	.106
25	64.84	.001^*∗∗∗*^	0.15	.001^*∗∗∗*^	30.23	.001^*∗∗∗*^	-0.38	.099	0.01	.997	0.07	.020^*∗*^
26	10.47	.005^*∗∗*^	-0.07	.001^*∗∗∗*^	2.13	.341	0.00	.512	28.34	.001^*∗∗∗*^	-0.07	.001^*∗∗∗*^
27	13.71	.001^*∗∗∗*^	0.00	.778	9.09	.010^*∗∗*^	-0.07	.924	0.85	.651	0.00	.914
28	18.73	.001^*∗∗∗*^	0.10	.001^*∗∗∗*^	18.70	.001^*∗∗∗*^	0.00	.590	0.79	.671	0.05	.003^*∗∗*^
29	9.31	.009^*∗∗*^	0.00	.047^*∗*^	10.32	.006^*∗∗*^	-0.43	.214	13.34	.001^*∗∗∗*^	0.00	.720
30	14.58	.001^*∗∗*^	-0.06	.008^*∗∗*^	14.57	.001^*∗∗∗*^	0.00	.403	3.83	.145	-0.07	.001^*∗∗∗*^
31	7.38	.024^*∗*^	0.02	.010^*∗∗*^	1.40	.493	-0.23	.687	2.57	.273	0.00	.108
32	18.09	.001^*∗∗∗*^	0.00	.028^*∗*^	0.65	.720	0.00	.908	6.31	.042^*∗*^	0.00	.422
33	15.01	.001^*∗∗∗*^	0.02	.005^*∗∗*^	0.40	.820	-0.14	.541	0.00	1.000	0.02	.006^*∗∗*^
34	30.39	.001^*∗∗∗*^	0.00	.963	1.61	.443	0.00	.937	6.57	.037^*∗*^	0.00	.823
35	9.62	.008^*∗∗*^	0.00	.606	0.37	.833	-0.26	.284	0.31	.859	0.02	.020^*∗*^
36	29.49	.001^*∗∗∗*^	-0.06	.016^*∗*^	1.11	.571	0.00	.357	5.88	.052	-0.02	.042^*∗*^

	Wave 4				Wave 5				Wave 6			
	Marital status		Urban and Regional		Marital status		Urban and Regional		Marital status		Urban and Regional	
ITEM	SUMMARY DIF	PROB.	DIF CONTRAST	Mantel- Haenszel Probability	SUMMARY DIF	PROB.	DIF CONTRAST	Mantel- Haenszel Probability	SUMMARY DIF	PROB.	DIF CONTRAST	Mantel- Haenszel Probability
No.	CHI-SQUARE (DIF=2)				CHI-SQUARE (DIF=2)				CHI-SQUARE (DIF=2)			

1	0.22	.898	0.00	.261	4.26	.117	0.14	.769	1.14	.560	0.11	.198
2	0.35	.841	0.00	.009^*∗∗*^	2.92	.229	0.00	.976	0.15	.930	0.00	.275
3	0.25	.882	0.00	.078	2.24	.323	-0.02	.403	5.55	.060	0.00	.613
4	0.03	.987	0.00	.145	1.97	.369	0.00	.756	4.67	.100	0.00	.027
5	0.66	.716	0.06	.001^*∗∗∗*^	0.46	.795	0.27	.618	1.40	.490	0.43	.083
6	6.42	.039^*∗*^	0.00	.555	5.78	.054	0.00	.271	11.47	.01^*∗∗∗*^	-0.14	.427
7	2.74	.251	0.00	.705	4.04	.130	-0.20	.574	8.64	.01^*∗∗*^	0.04	.948
8	1.19	.549	0.00	.165	0.87	.645	0.05	.039^*∗*^	1.17	.560	0.00	.117
9	4.04	.130	-0.04	.998	1.92	.379	-0.20	.371	3.26	.190	-0.04	.752
10	1.85	.392	0.00	.894	2.52	.280	0.11	.001^*∗∗∗*^	2.21	.330	0.10	.001^*∗∗∗*^
11	3.41	.179	0.00	.186	2.23	.324	-0.31	.823	1.92	.380	-0.08	.821
12	0.08	.965	0.00	.394	0.00	1.000	-0.02	.598	1.52	.460	0.00	.357
13	0.16	.927	0.00	.214	0.01	.998	0.01	.649	1.11	.570	-0.04	.916
14	0.03	.986	0.00	.368	1.12	.569	-0.06	.033^*∗*^	1.21	.540	-0.07	.274
15	3.06	.214	0.00	.611	0.00	1.000	-0.06	.860	0.86	.650	-0.09	.225
16	2.99	.221	0.00	.578	1.01	.602	0.00	.833	1.66	.430	0.00	.499
17	0.57	.753	0.00	.475	1.03	.594	-0.10	.754	0.64	.730	0.05	.290
18	0.08	.961	0.00	.671	4.27	.116	-0.07	.210	0.13	.940	-0.08	.987
19	0.11	.947	0.00	.420	2.78	.246	-0.08	.828	0.19	.910	-0.08	.986
20	0.36	.837	0.00	.089	5.27	.070	-0.05	.120	4.98	.080	-0.07	.758
21	1.67	.430	0.00	.169	1.16	.556	0.10	.439	0.21	.900	0.10	.046^*∗*^
22	0.54	.762	0.00	.049^*∗*^	2.89	.233	0.00	.446	1.95	.370	0.00	.874
23	21.23	.001^*∗∗∗*^	0.07	.002^*∗∗*^	0.50	.777	-0.07	.442	1.02	.600	0.00	.409
24	0.63	.730	0.00	.143	22.80	.001^*∗∗∗*^	0.00	.897	6.77	.030^*∗*^	0.02	.084
25	13.68	.001^*∗∗∗*^	0.00	.098	11.59	.003^*∗∗*^	0.00	.638	1.33	.510	-0.06	.169
26	0.41	.817	0.00	.021^*∗*^	8.24	.016^*∗*^	0.02	.274	1.17	.550	-0.03	.566
27	0.48	.787	0.00	.163	1.40	.494	0.22	.323	4.61	.100	0.18	.521
28	9.62	.008^*∗∗*^	0.00	.890	3.16	.203	-0.07	.109	0.04	.980	-0.11	.169
29	0.05	.979	-0.06	.008^*∗∗*^	4.42	.108	0.30	.161	2.07	.350	0.30	.104
30	2.04	.357	0.00	.035^*∗*^	6.85	.032^*∗*^	0.00	.859	1.79	.400	0.00	.517
31	0.47	.789	0.00	.068	6.88	.031^*∗*^	0.33	.165	0.00	1.000	0.12	.985
32	0.16	.923	0.00	.477	2.37	.302	0.00	.478	0.07	.970	-0.05	.889
33	3.33	.186	0.00	.180	5.40	.066	0.05	.851	0.45	.800	-0.20	.001^*∗∗∗*^
34	0.00	1.000	-0.03	.010^*∗*^	1.00	.605	0.00	.477	0.49	.780	0.00	.999
35	0.00	1.000	-0.04	.808	0.54	.764	0.27	.217	2.08	.350	-0.21	.006^*∗∗*^
36	2.74	.251	0.00	.065	5.82	.054	0.00	.495	1.44	.480	-0.03	.508

*Notes*. PROB. = probability; ^*∗*^*p* ≤ .05; ^*∗∗*^*p* ≤ .01; ^*∗∗∗*^*p* ≤ .001.

**Table 7:** SF-36 Physical health scale Rasch analysis item statistics for six waves of data collection.

	**Wave 1**			**Wave 2**			**Wave 3**		
**SF36 ITEM **	**MEASURE**	**MODEL** **S.E. **	**PTMEA** **CORR.**	**MEASURE**	**MODEL** **S.E. **	**PTMEA** **CORR.**	**MEASURE**	**MODEL** **S.E. **	**PTMEA** **CORR.**
**1:Q1**	-0.84	0.01	-0.21	-0.92	0.01	-0.24	-0.91	0.01	-0.18
**3:Q3A**	1.62	0.02	0.37	1.58	0.02	0.44	1.63	0.02	0.43
**4:Q3B**	0.02	0.01	0.59	0.00	0.01	0.61	0.13	0.01	0.60
**5:Q3C**	-0.05	0.01	0.59	-0.08	0.01	0.60	-0.08	0.01	0.59
**6:Q3D**	0.52	0.01	0.60	0.48	0.01	0.63	0.55	0.01	0.62
**7:Q3E**	-0.24	0.01	0.65	-0.27	0.01	0.67	-0.19	0.01	0.67
**8:Q3F**	0.21	0.01	0.57	0.26	0.01	0.58	0.33	0.01	0.54
**9:Q3G**	0.11	0.01	0.64	0.15	0.01	0.66	0.34	0.01	0.63
**10:Q3H**	-0.34	0.01	0.67	-0.34	0.01	0.68	-0.19	0.01	0.67
**11:Q3I**	-0.57	0.01	0.59	-0.62	0.01	0.59	-0.54	0.01	0.61
**12:Q3J**	-0.74	0.01	0.43	-0.84	0.01	0.40	-0.80	0.01	0.40
**13:Q4A**	0.96	0.01	0.42	0.95	0.01	0.41	1.02	0.02	0.38
**14:Q4B**	1.32	0.01	0.42	1.37	0.02	0.43	1.46	0.02	0.39
**15:Q4C**	1.16	0.01	0.46	1.17	0.01	0.50	1.20	0.02	0.42
**16:Q4D**	1.20	0.01	0.44	1.22	0.02	0.47	1.28	0.02	0.42
**21:Q7**	-0.74	0.01	-0.05	0.99	0.01	-0.19	-0.95	0.01	-0.02
**22:Q8**	0.36	0.01	-0.18	-0.78	0.01	-0.15	0.04	0.01	-0.14
**33:Q11A**	-2.22	0.01	0.34	-2.49	0.01	0.34	-2.46	0.01	0.32
**34:Q11B**	-0.07	0.01	0.02	0.04	0.01	-0.07	-0.09	0.01	0.02
**35:Q11C**	-1.24	0.01	0.38	-1.42	0.01	0.38	-1.27	0.01	0.37
**36:Q11D**	-0.42	0.01	-0.09	-0.45	0.01	-0.18	-0.50	0.01	-0.08

	**Wave 4**			**Wave 5**			**Wave 6**		
**SF36 ITEM **	**MEASURE**	**MODEL** **S.E. **	**PTMEA** **CORR.**	**MEASURE**	**MODEL** **S.E. **	**PTMEA** **CORR.**	**MEASURE**	**MODEL** **S.E. **	**PTMEA** **CORR.**

**1:Q1**	-1.10	0.01	-0.14	-1.21	0.01	-0.13	-1.32	0.02	-0.06
**3:Q3A**	1.64	0.02	0.42	1.63	0.03	0.42	1.79	0.04	0.39
**4:Q3B**	0.26	0.02	0.60	0.33	0.02	0.60	0.47	0.02	0.57
**5:Q3C**	0.02	0.01	0.60	0.05	0.02	0.58	0.09	0.02	0.56
**6:Q3D**	0.59	0.02	0.62	0.67	0.02	0.62	0.77	0.02	0.59
**7:Q3E**	-0.16	0.01	0.65	-0.09	0.02	0.65	-0.02	0.02	0.64
**8:Q3F**	0.32	0.02	0.57	0.30	0.02	0.57	0.34	0.02	0.53
**9:Q3G**	0.46	0.02	0.63	0.62	0.02	0.61	0.76	0.02	0.60
**10:Q3H**	-0.07	0.01	0.66	0.04	0.02	0.65	0.17	0.02	0.63
**11:Q3I**	-0.50	0.01	0.58	-0.43	0.02	0.58	-0.40	0.02	0.58
**12:Q3J**	-0.76	0.01	0.42	-0.75	0.01	0.41	-0.79	0.02	0.39
**13:Q4A**	1.01	0.02	0.37	1.03	0.02	0.34	0.99	0.03	0.34
**14:Q4B**	1.47	0.02	0.38	1.47	0.03	0.35	1.45	0.03	0.33
**15:Q4C**	1.27	0.02	0.43	1.29	0.02	0.40	1.29	0.03	0.35
**16:Q4D**	1.31	0.02	0.41	1.33	0.02	0.39	1.32	0.03	0.36
**21:Q7**	-1.08	0.01	-0.01	-1.17	0.01	0.01	-1.31	0.02	0.08
**22:Q8**	-0.19	0.01	-0.13	-0.35	0.01	-0.06	-0.53	0.02	-0.02
**33:Q11A**	-2.45	0.01	0.28	-2.43	0.02	0.26	-2.49	0.02	0.21
**34:Q11B**	-0.20	0.01	0.01	-0.38	0.02	0.06	-0.53	0.02	0.11
**35:Q11C**	-1.19	0.01	0.34	-1.08	0.01	0.33	-1.08	0.02	0.31
**36:Q11D**	-0.68	0.01	-0.09	-0.85	0.01	-0.04	-0.98	0.02	0.02

*Note*. MODEL S.E. = model standard error; PTMEA CORR = point measure correlation.

**Table 8:** SF-36 Physical health scale Rasch analysis Infit and Outfit statistics for six waves of data collection.

	**Wave 1 **				**Wave 2 **				**Wave 3 **			
**SF36 ITEM **	**Infit**		**Outfit**		**Infit**		**Outfit**		**Infit**		**Outfit**	
	**MNSQ**	**ZSTD**	**MNSQ**	**ZSTD**	**MNSQ**	**ZSTD**	**MNSQ**	**ZSTD**	**MNSQ**	**ZSTD**	**MNSQ**	**ZSTD**
**1:Q1**	1.24	9.9	1.30	9.9	1.24	9.9	1.29	9.9	1.16	9.9	1.20	9.9
**3:Q3A**	0.93	-4.6	0.90	-6.3	0.97	-1.8	0.90	-6.2	0.93	-3.7	0.85	-7.6
**4:Q3B**	0.57	-9.9	0.59	-9.9	0.59	-9.9	0.60	-9.9	0.64	-9.9	0.65	-9.9
**5:Q3C**	0.53	-9.9	0.54	-9.9	0.53	-9.9	0.54	-9.9	0.54	-9.9	0.56	-9.9
**6:Q3D**	0.72	-9.9	0.73	-9.9	0.71	-9.9	0.71	-9.9	0.72	-9.9	0.71	-9.9
**7:Q3E**	0.44	-9.9	0.46	-9.9	0.45	-9.9	0.47	-9.9	0.48	-9.9	0.50	-9.9
**8:Q3F**	0.62	-9.9	0.63	-9.9	0.64	-9.9	0.64	-9.9	0.67	-9.9	0.67	-9.9
**9:Q3G**	0.71	-9.9	0.72	-9.9	0.73	-9.9	0.75	-9.9	0.81	-9.9	0.81	-9.9
**10:Q3H**	0.45	-9.9	0.49	-9.9	0.50	-9.9	0.53	-9.9	0.59	-9.9	0.62	-9.9
**11:Q3I**	0.28	-9.9	0.32	-9.9	0.30	-9.9	0.33	-9.9	0.36	-9.9	0.39	-9.9
**12:Q3J**	0.21	-9.9	0.23	-9.9	0.18	-9.9	0.19	-9.9	0.21	-9.9	0.23	-9.9
**13:Q4A**	0.36	-9.9	0.40	-9.9	0.37	-9.9	0.40	-9.9	0.44	-9.9	0.48	-9.9
**14:Q4B**	0.51	-9.9	0.53	-9.9	0.53	-9.9	0.54	-9.9	0.59	-9.9	0.60	-9.9
**15:Q4C**	0.44	-9.9	0.47	-9.9	0.43	-9.9	0.45	-9.9	0.49	-9.9	0.52	-9.9
**16:Q4D**	0.46	-9.9	0.49	-9.9	0.46	-9.9	0.48	-9.9	0.52	-9.9	0.55	-9.9
**21:Q7**	*2.33*	9.9	*2.40*	9.9	*2.51*	9.9	*2.77*	9.9	*2.20*	9.9	*2.23*	9.9
**22:Q8**	*2.29*	9.9	*2.39*	9.9	*2.66*	9.9	*2.72*	9.9	*2.23*	9.9	*2.29*	9.9
**33:Q11A**	1.24	9.9	1.20	9.9	1.10	7.1	1.06	3.9	1.12	7.2	1.08	4.4
**34:Q11B**	*2.18*	9.9	*2.20*	9.9	*2.07*	9.9	*2.09*	9.9	*1.88*	9.9	*1.89*	9.9
**35:Q11C**	1.25	9.9	1.26	9.9	1.16	9.9	1.17	9.9	1.17	9.9	1.18	9.9
**36:Q11D**	*2.21*	9.9	*2.28*	9.9	*2.36*	9.9	*2.41*	9.9	*2.12*	9.9	*2.15*	9.9

	**Wave 4 **				**Wave 5**				**Wave 6**			
**SF36 ITEM **	**Infit**		**Outfit**		**Infit**		**Outfit**		**Infit**		**Outfit**	
	**MNSQ**	**ZSTD**	**MNSQ**	**ZSTD**	**MNSQ**	**ZSTD**	**MNSQ**	**ZSTD**	**MNSQ**	**ZSTD**	**MNSQ**	**ZSTD**

**1:Q1**	1.02	1.4	1.04	2.9	0.99	-0.8	1.00	0.0	0.92	-4.1	0.93	-3.4
**3:Q3A**	0.98	-0.9	0.87	-5.2	1.04	1.5	0.92	-2.8	1.12	3.1	0.95	-1.1
**4:Q3B**	0.67	-9.9	0.67	-9.9	0.69	-9.9	0.69	-9.9	0.76	-9.9	0.74	-9.9
**5:Q3C**	0.56	-9.9	0.57	-9.9	0.57	-9.9	0.58	-9.9	0.62	-9.9	0.63	-9.9
**6:Q3D**	0.72	-9.9	0.71	-9.9	0.76	-9.9	0.73	-9.9	0.80	-8.2	0.74	-9.9
**7:Q3E**	0.49	-9.9	0.51	-9.9	0.53	-9.9	0.55	-9.9	0.59	-9.9	0.60	-9.9
**8:Q3F**	0.63	-9.9	0.64	-9.9	0.62	-9.9	0.62	-9.9	0.65	-9.9	0.66	-9.9
**9:Q3G**	0.83	-9.9	0.81	-9.9	0.86	-7.0	0.83	-8.8	0.90	-3.9	0.83	-6.7
**10:Q3H**	0.66	-9.9	0.68	-9.9	0.72	-9.9	0.73	-9.9	0.79	-9.4	0.79	-9.5
**11:Q3I**	0.39	-9.9	0.42	-9.9	0.46	-9.9	0.48	-9.9	0.50	-9.9	0.53	-9.9
**12:Q3J**	0.28	-9.9	0.30	-9.9	0.33	-9.9	0.35	-9.9	0.37	-9.9	0.40	-9.9
**13:Q4A**	0.47	-9.9	0.51	-9.9	0.53	-9.9	0.58	-9.9	0.55	-9.9	0.59	-9.9
**14:Q4B**	0.62	-9.9	0.63	-9.9	0.65	-9.9	0.66	-9.9	0.68	-9.9	0.69	-9.9
**15:Q4C**	0.54	-9.9	0.57	-9.9	0.59	-9.9	0.61	-9.9	0.63	-9.9	0.65	-9.9
**16:Q4D**	0.56	-9.9	0.59	-9.9	0.60	-9.9	0.62	-9.9	0.63	-9.9	0.65	-9.9
**21:Q7**	*2.08*	9.9	*2.09*	9.9	*1.96*	9.9	*1.96*	9.9	*1.79*	9.9	*1.79*	9.9
**22:Q8**	*2.07*	9.9	*2.11*	9.9	*1.95*	9.9	*1.97*	9.9	*1.85*	9.9	*1.86*	9.9
**33:Q11A**	1.13	7.2	1.11	5.6	1.13	6.3	1.11	5.1	1.18	7.2	1.20	7.5
**34:Q11B**	*1.85*	9.9	*1.86*	9.9	*1.69*	9.9	*1.69*	9.9	*1.62*	9.9	*1.59*	9.9
**35:Q11C**	1.18	9.9	1.19	9.9	1.15	8.0	1.16	8.4	1.13	5.9	1.13	6.1
**36:Q11D**	*2.05*	9.9	*2.09*	9.9	*1.95*	9.9	*1.96*	9.9	*1.83*	9.9	*1.82*	9.9

*Notes*. MNSQ = mean square residual fit statistic; ZSTD: standardized mean square residual fit statistic; values in italic for Infit or Outfit MnSq > 1.34; values underlined for Infit or Outfit MnSq < 0.64.

**Table 9:** SF-36 physical health scale Rasch analysis summary Item and Person Infit and Outfit statistics for six waves of data collection.

		**Wave 1 **	**Wave 2 **	**Wave 3**	**Wave 4 **	**Wave 5 **	**Wave 6 **
**Persons**	**MEAN**	-1.77	-1.85	-1.90	-1.92	-1.95	-2.07
	**S.D.**	.38	.37	.40	.39	.39	.40
	**MAX**	1.54	-.37	.40	.92	-.52	.40
	**MIN**	-5.13	-4.11	-.09	-5.08	-4.52	-.79
	**Infit-MNSQ**	1.05	1.04	1.05	1.05	1.05	1.05
	**Infit-ZSTD**	-.10	-.20	-.10	.00	.00	.00
	**Outfit-MNSQ**	1.00	1.01	.98	.97	.96	.96
	**Outfit-ZSTD**	-.30	-.30	-.30	-.20	-.20	-.20
	**Person separation**	.86^c^	.88^c^	.97^c^	.96^c^	.96^c^	.96^c^
	**Person reliability**	.43^a^	.43^a^	.48^a^	.48^a^	.48^a^	.48^a^

**Items**	**MEAN**	.00	.00	.00	.00	.00	.00
	**S.D.**	.91	.99	.98	1.00	1.02	1.08
	**MAX**	1.62	1.58	1.63	1.64	1.63	1.79
	**MIN**	-2.22	-2.49	-2.46	-2.45	-2.43	-2.49
	**Infit-MNSQ**	.95	.98	.95	.94	.94	.95
	**Infit-ZSTD**	-3.00	-3.00	-3.10	-3.40	-3.40	-3.30
	**Outfit-MNSQ**	.98	1.00	.96	.95	.94	.94
	**Outfit-ZSTD**	-3.10	-3.40	-3.50	-3.60	-3.70	-3.60
	**Item separation**	71.24	69.37	63.25	59.41	52.87	45.77
	**Item reliability**	1.00	1.00	1.00	1.00	1.00	1.00

*Notes*. ^a^Person or item reliability <0.8; ^b^Item separation <3.0; ^c^Person separation <2.0; values in italic for Infit or Outfit MnSq > 1.34; values underlined for Infit or Outfit MnSq < 0.64.

**Table 10:** SF-36 physical health scale Rasch analysis of standardised residual variance in Eigenvalue units for six waves of data collection.

	**Wave 1**			**Wave 2 **			**Wave 3**		
	**Eigenvalue**	**Observed**	**Expected**	**Eigenvalue**	**Observed**	**Expected**	**Eigenvalue**	**Observed**	**Expected**
**Total raw variance in observations**	36.07	100.00%	100.00%	36.07	100.00%	100.00%	37.89	100.00%	100.00%

**Raw variance explained by measures**	15.07	41.80%	42.50%	15.07	41.80%	42.50%	16.89	44.60%	45.70%

**Raw variance explained by persons**	1.90	5.30%	5.40%	1.90	5.30%	5.40%	0.96	2.50%	2.60%

**Raw Variance explained by items**	13.16	36.50%	37.10%	13.16	36.50%	37.10%	15.94	42.10%	43.10%

**Raw unexplained variance (total)**	21.00	58.20%	57.50%	21.00	58.20%	57.50%	21.00	55.40%	54.30%

**Unexplained variance in 1st contrast**	8.00	22.20%	38.10%	8.00	22.20%	38.10%	7.70	20.30%	36.70%

**Unexplained variance in 2nd contrast**	2.02	5.60%	9.60%	2.02	5.60%	9.60%	1.96	5.20%	9.40%

**Unexplained variance in 3rd contrast**	1.51	4.20%	7.20%	1.51	4.20%	7.20%	1.44	3.80%	6.90%

**Unexplained variance in 4th contrast**	1.31	3.60%	6.20%	1.31	3.60%	6.20%	1.23	3.20%	5.80%

**Unexplained variance in 5th contrast**	0.99	2.80%	4.70%	0.99	2.80%	4.70%	0.99	2.60%	4.70%

	**Wave 4**			**Wave 5**			**Wave 6**		
	**Eigenvalue**	**Observed**	**Expected**	**Eigenvalue**	**Observed**	**Expected**	**Eigenvalue**	**Observed**	**Expected**

**Total raw variance in observations**	37.07	100.00%	100.00%	39.34	100.00%	100.00%	41.08	100.00%	100.00%

**Raw variance explained by measures**	17.07	46.10%	48.00%	18.34	46.60%	48.60%	20.08	48.90%	51.10%

**Raw variance explained by persons**	2.45	6.60%	6.90%	2.42	6.10%	6.40%	2.68	6.50%	6.80%

**Raw Variance explained by items**	14.62	39.40%	41.10%	15.92	40.50%	42.20%	17.40	42.30%	44.20%

**Raw unexplained variance (total)**	20.00	53.90%	52.00%	21.00	53.40%	51.40%	21.00	51.10%	48.90%

**Unexplained variance in 1st contrast**	6.64	17.90%	33.20%	7.50	19.10%	35.70%	7.14	17.40%	34.00%

**Unexplained variance in 2nd contrast**	2.10	5.70%	10.50%	2.06	5.20%	9.80%	2.24	5.50%	10.70%

**Unexplained variance in 3rd contrast**	1.54	4.20%	7.70%	1.58	4.00%	7.50%	1.56	3.80%	7.40%

**Unexplained variance in 4th contrast**	1.26	3.40%	6.30%	1.21	3.10%	5.80%	1.20	2.90%	5.70%

**Unexplained variance in 5th contrast**	1.07	2.90%	5.30%	1.03	2.60%	4.90%	1.04	2.50%	4.90%

*Notes.* ^a^ > 60% unexplained variance in the Rasch factor; ^b^Eigenvalue in the first contrast <3.0; ^c^ < 10% unexplained variance in the first contrast.

**Table 11:** SF-36 physical health scale Rasch analysis of summary of category structure for six waves of data collection.

	**WAVE 1**					**WAVE 2**						**WAVE 3**						
**CAT.** **LABEL**	**N**	%	**Average Measures**	**Infit MnSq**	**Outfit MnSq**	**Andrich Thresholds**	**N**	%	**Average Measures**	**Infit MnSq**	**Outfit MnSq**	**Andrich Thresholds**	**N**	%	**Average Measures**	**Infit MnSq**	**Outfit MnSq**	**Andrich Thresholds**
1	60721	23	(-3.86)	1.18	1.11	NONE	55350	25	( -3.92)	1.12	1.07	NONE	46995	28	( -3.86)	1.14	1.08	NONE
2	83039	32	-2.13	.93	.94	-2.56	70454	32	-2.19	.93	.92	-2.60	54692	32	-2.17	1.00	.99	-2.54
3	73299	28	-.83	.66	.59	-1.54	62780	29	-.85	.66	.60	-1.61	46905	28	-.90	.72	.62	-1.61
4	12957	5	.10	1.19	1.26	.59	11389	5	.14	1.21	*1.38*	.53	9720	6	.07	1.11	1.11	.38
5	15144	6	1.96	1.05	1.15	-.71^b^	12634	6	2.03	1.13	1.33	-.55^b^	9942	6	2.05	1.08	1.17	-.56^b^
6	238	0	(5.32)	*2.67*	*2.61*	4.21	233	0	(5.34)	*3.18*	*2.98*	4.23	155	0	(5.43)	*2.77*	*2.30*	4.33

	**WAVE 4**						**WAVE 5**						**WAVE 6**					
**CAT.** **LABEL**	**N**	**%**	**Average Measures**	**Infit MnSq**	**Outfit MnSq**	**Andrich Thresholds**	**N**	**%**	**Average Measures**	**Infit MnSq**	**Outfit MnSq**	**Andrich Thresholds**	**N**	**%**	**Average Measures**	**Infit MnSq**	**Outfit MnSq**	**Andrich Thresholds**

1	42924	29	( -3.75)	1.15	1.08	NONE	36502	31	( -3.66)	1.15	1.08	NONE	28787	36	( -3.64)	1.15	1.08	NONE
2	44603	30	-2.09	1.05	1.02	-2.42	33751	29	-2.01	1.08	1.01	-2.33	23233	29	-2.02	1.10	1.01	-2.30
3	36071	24	-.89	.76	.64	-1.52	25389	22	-.87	.82	.68	-1.44	16930	21	-.91	.86	.73	-1.46
4	8958	6	.06	1.00	.96	.24	7310	6	.06	.94	.86	.13	5353	7	.01	.91	.80	.03
5	8592	6	2.01	1.10	1.16	-.48^b^	6464	6	1.96	1.09	1.12	-.38^b^	4694	6	2.00	1.09	1.10	-.39^b^
6	150	0	(5.29)	*2.54*	*2.02*	4.18	129	0	(5.12)	*2.25*	*1.75*	4.02	80	0	(5.24)	*2.03*	*1.52*	4.13

*Notes.* ^a^Andrich threshold category increase of >5; ^b^Andrich threshold category decrease where an increase is expected; values in italic for Infit or Outfit MnSq > 1.34; values underlined for Infit or Outfit MnSq < 0.64.

**Table 12:** Differential Item Functioning (DIF) for SF-36 physical health scale Rasch analysis for six waves of data collection based on marital status and area of residence.

	**Wave 1**				**Wave 2 **				**Wave 3**			
	**Marital status**		**Urban and Regional**		**Marital status**		**Urban and Regional**		**Marital status**		**Urban and Regional**	
**SF36 ITEM** **No.**	**SUM DIF** **CHI-SQUARE (DIF = 2)**	**PROB.**	**DIF CONTRAST**	**Mantel- Haenszel Probability**	**SUM DIF** **CHI-SQUARE (DIF = 2)**	**PROB**	**DIF CONTRAST**	**Mantel- Haenszel Probability**	**SUM DIF** **CHI-SQUARE (DIF = 2)**	**PROB**	**DIF CONTRAST**	**Mantel- Haenszel Probability**
**1:Q1**	13.25	.001^*∗∗∗*^	0.00	.639	1.62	.442	0.14	.713	0.41	.816	0.00	.185
**3:Q3A**	5.79	.054	0.00	.725	1.27	.527	0.00	.330	5.69	.057	0.00	.073
**4:Q3B**	1.94	.376	0.00	.069	0.56	.754	0.15	.835	0.50	.779	0.06	.001^*∗∗∗*^
**5:Q3C**	1.84	.394	0.06	.001^*∗∗∗*^	0.03	.984	0.00	.009^*∗∗*^	0.56	.756	0.00	.442
**6:Q3D**	0.97	.614	0.00	.947	2.13	.342	-0.20	.778	0.44	.804	0.00	.700
**7:Q3E**	0.41	.816	0.00	.287	2.75	.250	0.00	.153	1.26	.529	0.00	.143
**8:Q3F**	0.06	.970	0.00	.684	0.18	.917	-0.08	.599	0.03	.988	-0.04	.964
**9:Q3G**	13.16	.001^*∗∗∗*^	-0.02	.076	1.33	.512	0.03	.006^*∗∗*^	0.57	.750	0.00	.847
**10:Q3H**	12.78	.002^*∗∗*^	0.00	.324	5.72	.056	-0.22	.320	0.02	.990	0.00	.225
**11:Q3I**	7.45	.024^*∗*^	0.00	.357	5.55	.061	0.07	.001^*∗∗∗*^	0.00	1.000	0.00	.631
**12:Q3J**	0.95	.620	0.00	.306	0.03	.988	-0.03	.836	0.73	.693	0.00	.251
**13:Q4A**	2.34	.306	0.00	.519	0.08	.962	0.00	.461	0.17	.919	0.00	.360
**14:Q4B**	1.45	.481	0.00	.782	4.22	.119	-0.30	.206	6.61	.036^*∗*^	0.00	.520
**15:Q4C**	2.47	.288	0.00	.982	0.08	.961	0.00	.240	0.37	.831	0.00	.524
**16:Q4D**	0.08	.965	0.00	.845	0.06	.973	0.00	.873	4.54	.101	0.00	.053
**21:Q7**	2.54	.277	-0.05	.005^*∗∗*^	9.34	.009^*∗∗*^	0.00	.131	0.13	.941	0.00	.145
**22:Q8**	37.29	.001^*∗∗∗*^	0.00	.114	1.00	.605	-0.09	.651	1.85	.394	0.02	.081
**33:Q11A**	27.3	.001^*∗∗∗*^	0.07	.001^*∗∗∗*^	1.11	.572	0.00	.521	0.02	.990	0.00	.275
**34:Q11B**	36.38	.001^*∗∗∗*^	0.00	.905	1.41	.490	-0.20	.309	6.66	.035^*∗*^	0.00	.170
**35:Q11C**	12.2	.002^*∗∗*^	0.00	.204	0.68	.710	0.00	.963	0.58	.749	-0.05	.002^*∗∗*^
**36:Q11D**	35.29	.001^*∗∗∗*^	-0.05	.006^*∗∗*^	1.38	.500	0.12	.444	7.03	.029^*∗*^	0.00	.724

	**Wave 4**				**Wave 5**				**Wave 6**			
	**Marital status **		**Urban and Regional**		**Marital status**		**Urban and Regional**		**Marital status**		**Urban and Regional**	
**SF36 ITEM** **No.**	**SUM DIF** **CHI-SQUARE (DIF = 2)**	**PROB**	**DIF CONTRAST**	**Mantel- Haenszel Probability**	**SUM DIF** **CHI-SQUARE (DIF = 2)**	**PROB**	**DIF CONTRAST**	**Mantel- Haenszel Probability**	**SUM DIF** **CHI-SQUARE (DIF = 2)**	**PROB**	**DIF CONTRAST**	**Mantel- Haenszel Probability**

**1:Q1**	0.67	.714	0.00	.185	4.44	.107	0.23	.707	2.87	.235	0.09	.418
**3:Q3A**	0.22	.897	0.00	.073	1.32	.515	0.00	.375	3.73	.153	0.00	.176
**4:Q3B**	0.26	.878	0.06	.001^*∗∗∗*^	0.59	.744	0.34	.229	2.71	.254	0.38	.098
**5:Q3C**	3.48	.173	0.00	.442	0.65	.720	0.00	.342	1.81	.400	-0.13	.270
**6:Q3D**	1.39	.496	0.00	.700	2.83	.240	-0.16	.573	7.85	.019^*∗*^	0.00	.761
**7:Q3E**	0.10	.953	0.00	.143	1.73	.418	0.03	.025^*∗*^	6.17	.045^*∗*^	0.00	.278
**8:Q3F**	2.31	.311	-0.04	.964	0.00	1.000	-0.17	.456	0.43	.808	-0.08	.248
**9:Q3G**	0.95	.621	0.00	.847	0.39	.824	0.12	.001^*∗∗∗*^	1.20	.547	0.11	.001^*∗∗∗*^
**10:Q3H**	1.89	.384	0.00	.225	0.73	.695	-0.26	.443	0.68	.712	-0.12	.739
**11:Q3I**	0.00	1.000	0.00	.631	0.42	.809	0.00	.961	0.55	.761	0.00	.387
**12:Q3J**	0.05	.975	0.00	.251	0.10	.953	0.06	.252	0.65	.722	-0.03	.664
**13:Q4A**	0.45	.798	0.00	.360	0.00	1.000	-0.06	.042^*∗*^	0.17	.922	-0.07	.282
**14:Q4B**	1.60	.447	0.00	.520	1.98	.367	-0.05	.861	0.82	.663	-0.12	.138
**15:Q4C**	2.50	.283	0.00	.524	0.01	.996	0.00	.453	0.20	.908	0.00	.255
**16:Q4D**	0.68	.711	0.00	.053	0.24	.889	-0.06	.733	0.73	.692	0.02	.431
**21:Q7**	3.61	.162	0.00	.145	0.00	1.000	-0.06	.413	1.75	.413	-0.07	.650
**22:Q8**	1.03	.595	0.02	.081	3.03	.217	0.00	.310	4.23	.119	-0.10	.644
**33:Q11A**	0.14	.934	0.00	.275	13.77	.001^*∗∗∗*^	-0.05	.170	1.34	.509	-0.07	.729
**34:Q11B**	4.03	.131	0.00	.170	1.80	.403	0.20	.048^*∗*^	2.28	.317	0.07	.252
**35:Q11C**	0.00	1.000	-0.05	.002^*∗∗*^	0.49	.783	0.00	.280	3.68	.156	0.00	.681
**36:Q11D**	0.37	.831	0.00	.724	7.48	.023^*∗*^	0.00	.941	1.55	.457	-0.03	.897

*Notes*. PROB. = probability; ^*∗*^*p* ≤ .05; ^*∗∗*^*p* ≤ .01; ^*∗∗∗*^*p* ≤ .001.

**Table 13:** SF-36 mental health scale Rasch analysis item statistics for six waves of data collection.

	**Wave 1**			**Wave 2**			**Wave 3**		
**SF36 ITEM **	**MEASURE**	**MODEL S.E. **	**PTMEA CORR.**	**MEASURE**	**MODEL S.E. **	**PTMEA CORR.**	**MEASURE**	**MODEL S.E. **	**PTMEA CORR**
**17:Q5A**	1.35	0.01	0.31	1.38	0.01	0.30	1.49	0.02	0.27
**18:Q5B**	1.57	0.01	0.31	1.62	0.02	0.29	1.75	0.02	0.27
**19:Q5C**	1.38	0.01	0.30	1.41	0.01	0.28	1.50	0.02	0.26
**20:Q6**	1.51	0.01	-0.09	1.78	0.02	-0.02	1.41	0.01	-0.02
**23:Q9A**	-0.03	0.01	0.17	-0.06	0.01	0.22	-0.12	0.01	0.27
**24:Q9B**	-1.28	0.01	0.46	-1.47	0.01	0.43	-1.54	0.01	0.41
**25:Q9C**	-1.84	0.01	0.45	-2.04	0.01	0.40	-2.08	0.02	0.40
**26:Q9D**	0.21	0.01	0.20	0.30	0.01	0.18	0.30	0.01	0.26
**27:Q9E**	-0.16	0.01	0.22	-0.24	0.01	0.26	-0.29	0.01	0.30
**28:Q9F**	-1.25	0.01	0.46	-1.33	0.01	0.39	-1.32	0.01	0.40
**29:Q9G**	-0.90	0.01	0.44	-0.93	0.01	0.39	-0.88	0.01	0.39
**30:Q9H**	0.63	0.01	0.27	0.72	0.01	0.25	0.74	0.01	0.28
**31:Q9I**	-0.55	0.01	0.37	-0.50	0.01	0.31	-0.42	0.01	0.31
**32:Q10**	-0.65	0.01	0.28	-0.65	0.01	0.22	-0.55	0.01	0.19

	**Wave 4 **			**Wave 5**			**Wave 6**		
**SF36 ITEM **	**MEASURE**	**MODEL S.E. **	**PTMEA CORR**	**MEASURE**	**MODEL S.E. **	**PTMEA CORR**	**MEASURE**	**MODEL S.E. **	**PTMEA CORR.**

**17:Q5A**	1.47	0.02	0.28	1.48	0.02	0.30	1.51	0.02	0.29
**18:Q5B**	1.76	0.02	0.28	1.77	0.02	0.30	1.81	0.03	0.32
**19:Q5C**	1.51	0.02	0.27	1.51	0.02	0.28	1.53	0.02	0.28
**20:Q6**	1.19	0.01	0.04	1.01	0.02	0.03	0.96	0.02	0.05
**23:Q9A**	-0.14	0.01	0.23	-0.21	0.01	0.24	-0.29	0.01	0.26
**24:Q9B**	-1.52	0.01	0.40	-1.49	0.02	0.40	-1.47	0.02	0.37
**25:Q9C**	-2.07	0.02	0.35	-1.92	0.02	0.39	-1.91	0.02	0.35
**26:Q9D**	0.30	0.01	0.23	0.31	0.01	0.19	0.29	0.01	0.22
**27:Q9E**	-0.34	0.01	0.27	-0.41	0.01	0.27	-0.53	0.01	0.29
**28:Q9F**	-1.30	0.01	0.40	-1.25	0.01	0.42	-1.22	0.02	0.41
**29:Q9G**	-0.80	0.01	0.39	-0.75	0.01	0.44	-0.72	0.01	0.43
**30:Q9H**	0.75	0.01	0.29	0.69	0.01	0.23	0.69	0.02	0.27
**31:Q9I**	-0.34	0.01	0.33	-0.30	0.01	0.35	-0.27	0.01	0.32
**32:Q10**	-0.48	0.01	0.17	-0.44	0.01	0.17	-0.39	0.01	0.17

*Note*: MODEL S.E. = Model Standard Error; PTMEA CORR = Point Measure Correlation.

**Table 14:** SF-36 mental health scale Rasch analysis Infit and Outfit statistics for six waves of data collection.

	**Wave 1**				**Wave 2**				**Wave 3**			
**SF36 ITEM **	**Infit**		**Outfit**		**Infit**		**Outfit**		**Infit**		**Outfit**	
	**MNSQ**	**ZSTD**	**MNSQ**	**ZSTD**	**MNSQ**	**ZSTD**	**MNSQ**	**ZSTD**	**MNSQ**	**ZSTD**	**MNSQ**	**ZSTD**
**17:Q5A**	0.29	-9.9	0.32	-9.9	0.26	-9.9	0.28	-9.9	0.30	-9.9	0.32	-9.9
**18:Q5B**	0.43	-9.9	0.46	-9.9	0.43	-9.9	0.46	-9.9	0.48	-9.9	0.50	-9.9
**19:Q5C**	0.31	-9.9	0.34	-9.9	0.29	-9.9	0.31	-9.9	0.32	-9.9	0.34	-9.9
**20:Q6**	*2.64*	9.9	*2.87*	9.9	*2.91*	9.9	*3.06*	9.9	*2.54*	9.9	*2.74*	9.9
**23:Q9A**	1.08	7.7	1.15	9.9	1.03	3.2	1.12	9.9	1.04	3.5	1.09	6.7
**24:Q9B**	1.25	9.9	1.17	9.9	1.40	9.9	1.29	9.9	*1.41*	9.9	1.31	9.9
**25:Q9C**	*1.44*	9.9	1.30	9.9	*1.59*	9.9	*1.39*	*9.9*	*1.51*	9.9	1.33	9.9
**26:Q9D**	1.22	9.9	1.33	9.9	1.14	9.9	1.28	9.9	1.10	7.1	1.19	9.9
**27:Q9E**	1.12	9.9	1.17	9.9	1.08	7.8	1.12	9.9	1.07	5.3	1.08	6.3
**28:Q9F**	0.90	-6.6	0.88	-8.3	1.02	1.0	0.98	-0.9	0.96	-2.0	0.93	-3.6
**29:Q9G**	0.88	-9.9	0.87	-9.9	0.90	-7.4	0.89	-7.8	0.88	-7.8	0.87	-8.2
**30:Q9H**	1.22	9.9	1.29	9.9	1.15	8.5	1.24	9.9	1.09	4.6	1.16	7.9
**31:Q9I**	0.72	-9.9	0.73	-9.9	0.76	-9.9	0.77	-9.9	0.73	-9.9	0.74	-9.9
**32:Q10**	0.72	-9.9	0.77	-9.9	0.78	-9.9	0.81	-9.9	0.87	-9.9	0.91	-6.8

	**Wave 4**				**Wave 5**				**Wave 6**			
**SF36 ITEM**	**Infit**		**Outfit**		**Infit**		**Outfit**		**Infit**		**Outfit**	
	**MNSQ**	**ZSTD**	**MNSQ**	**ZSTD**	**MNSQ**	**ZSTD**	**MNSQ**	**ZSTD**	**MNSQ**	**ZSTD**	**MNSQ**	ZSTD

**17:Q5A**	0.31	-9.9	0.34	-9.9	0.34	-9.9	0.37	-9.9	0.36	-9.9	0.39	-9.9
**18:Q5B**	0.50	-9.9	0.52	-9.9	0.52	-9.9	0.54	-9.9	0.53	-9.9	0.55	-9.9
**19:Q5C**	0.34	-9.9	0.36	-9.9	0.37	-9.9	0.39	-9.9	0.38	-9.9	0.41	-9.9
**20:Q6**	*2.16*	9.9	*2.33*	9.9	*1.96*	9.9	*2.15*	9.9	*1.91*	9.9	*2.07*	9.9
**23:Q9A**	1.04	2.7	1.07	5.3	1.02	1.7	1.05	3.4	1.03	1.8	1.04	2.2
**24:Q9B**	*1.37*	9.9	1.25	9.9	*1.36*	9.9	1.25	9.5	1.33	9.9	1.25	8.3
**25:Q9C**	*1.50*	9.9	*1.36*	9.9	*1.51*	9.9	*1.36*	9.9	*1.42*	9.9	1.32	9.1
**26:Q9D**	1.15	9.3	1.23	9.9	1.16	8.7	1.26	9.9	1.13	6.2	1.20	8.8
**27:Q9E**	1.11	7.9	1.11	8.0	1.08	5.1	1.08	4.8	1.10	5.2	1.09	4.4
**28:Q9F**	0.97	-1.6	0.94	-3.2	0.95	-2.2	0.91	-4.2	0.91	-3.5	0.89	-4.4
**29:Q9G**	0.87	-8.1	0.86	-8.5	0.84	-9.2	0.83	-9.7	0.85	-7.3	0.84	-7.8
**30:Q9H**	1.12	5.6	1.16	7.3	1.13	5.8	1.22	9.3	1.09	3.6	1.15	5.3
**31:Q9I**	0.76	-9.9	0.77	-9.9	0.76	-9.9	0.77	-9.9	0.76	-9.9	0.78	-9.9
**32:Q10**	0.86	-9.9	0.91	-6.9	0.90	-6.3	0.94	-3.9	0.96	-2.4	1.00	0.2

*Notes*. MNSQ = mean square residual fit statistic; ZSTD: standardized mean square residual fit statistic; values in italic for Infit or Outfit MnSq > 1.34; values underlined for Infit or Outfit MnSq < 0.64.

**Table 15:** SF-36 mental health scale Rasch analysis summary Item and Person Infit and Outfit statistics for six waves of data collection.

		**Wave 1**	**Wave 2**	**Wave 3**	**Wave 4**	**Wave 5**	**Wave 6**
**Persons**	**MEAN**	-.08	-.04	-.02	-.03	-.06	-.06
	**S.D.**	.30	.28	.31	.30	.29	.30
	**MAX**	.30	1.64	1.38	.30	.29	.30
	**MIN**	1.87	-3.54	-2.99	2.37	2.16	2.12
	**Infit-MNSQ**	1.01	1.01	1.02	1.02	1.02	1.02
	**Infit-ZSTD**	-.30	-.40	-.30	-.30	-.30	-.20
	**Outfit-MNSQ**	1.06	1.08	1.06	1.03	1.02	1.01
	**Outfit-ZSTD**	-.20	-.20	-.20	-.20	-.20	-.20
	**Person separation**	.53	.33	.45	.36	.36	.41
	**Person reliability**	.22^a^	.10^a^	.17^a^	.11^a^	.12^a^	.14^a^

**Items**	**MEAN**	.00	.00	.00	.00	.00	.00
	**S.D.**	1.17	1.20	1.19	1.13	1.31	1.07
	**MAX**	1.59	1.78	1.75	1.75	2.13	1.67
	**MIN**	-1.88	-2.04	-2.08	-2.04	-1.94	-1.95
	**Infit-MNSQ**	1.02	1.05	1.02	1.00	.99	.98
	**Infit-ZSTD**	.10	.20	-.60	-.30	-.50	-.50
	**Outfit-MNSQ**	1.05	1.07	1.04	1.02	1.01	1.00
	**Outfit-ZSTD**	.10	.80	.20	.10	-.10	-.30
	**Item separation**	95.77	89.12	83.98	77.85	68.89	59.38
	**Item reliability**	1.00	1.00	1.00	1.00	1.00	1.00

*Notes*. ^a^Person or item reliability <0.8; ^b^Item separation <3.0; ^c^Person separation <2.0; values in italic for Infit or Outfit MnSq > 1.34; values underlined for Infit or Outfit MnSq < 0.64.

**Table 16:** SF-36 mental health scale Rasch analysis of standardised residual variance in Eigenvalue units for six waves of data collection.

	**WAVE 1**			**WAVE 2**			**WAVE 3**		
	**Eigenvalue**	**Observed**	**Expected**	**Eigenvalue**	**Observed**	**Expected**	**Eigenvalue**	**Observed**	**Expected**
**Total raw variance in observations**	38.55	100.00%	100.00%	41.29	100.00%	100.00%	42.62	100.00%	100.00%
**Raw variance explained by measures**	24.55	63.70%	63.70%	27.29	66.10%	66.20%	26.62	62.50%	62.50%
**Raw variance explained by persons**	2.85	7.40%	7.40%	2.06	5.00%	5.00%	2.68	6.30%	6.30%
**Raw Variance explained by items**	21.70	56.30%	56.30%	25.23	61.10%	61.20%	23.94	56.20%	56.20%
**Raw unexplained variance (total)**	14.00	36.30%	36.30%	14.00	33.90%	33.80%	16.00	37.50%	37.50%
**Unexplained variance in 1st contrast**	6.22	16.10%	44.50%	6.22	15.10%	44.40%	7.02	16.50%	43.90%
**Unexplained variance in 2nd contrast**	1.49	3.90%	10.60%	1.47	3.60%	10.50%	1.62	3.80%	10.10%
**Unexplained variance in 3rd contrast**	1.29	3.30%	9.20%	1.32	3.20%	9.40%	1.29	3.00%	8.10%
**Unexplained variance in 4th contrast**	0.81	2.10%	5.80%	0.85	2.00%	6.00%	1.05	2.50%	6.60%
**Unexplained variance in 5th contrast**	0.68	1.80%	4.90%	0.71	1.70%	5.00%	0.71	1.70%	4.40%

	**WAVE 4**			**WAVE 5**			**WAVE 6**		
	**Eigenvalue**	**Observed**	**Expected**	**Eigenvalue**	**Observed**	**Expected**	**Eigenvalue**	**Observed**	**Expected**

**Total raw variance in observations**	39.19	100.00%	100.00%	37.79	100.00%	100.00%	37.65	100.00%	100.00%
**Raw variance explained by measures**	25.19	64.30%	64.50%	23.79	62.90%	63.30%	23.65	62.80%	63.20%
**Raw variance explained by persons**	2.43	6.20%	6.20%	1.73	4.60%	4.60%	2.44	6.50%	6.50%
**Raw Variance explained by items**	22.76	58.10%	58.30%	22.06	58.40%	58.70%	21.21	56.30%	56.60%
**Raw unexplained variance (total)**	14.00	35.70%	35.50%	14.00	37.10%	36.70%	14.00	37.20%	36.80%
**Unexplained variance in 1st contrast**	6.16	15.70%	44.00%	6.10	16.10%	43.60%	5.75	15.30%	41.10%
**Unexplained variance in 2nd contrast**	1.52	3.90%	10.90%	1.61	4.20%	11.50%	1.67	4.40%	11.90%
**Unexplained variance in 3rd contrast**	1.32	3.40%	9.40%	1.31	3.50%	9.30%	1.35	3.60%	9.60%
**Unexplained variance in 4th contrast**	0.80	2.00%	5.70%	0.79	2.10%	5.60%	0.85	2.30%	6.10%
**Unexplained variance in 5th contrast**	0.68	1.70%	4.90%	0.69	1.80%	4.90%	0.68	1.80%	4.80%

*Notes*. ^a^ > 60% unexplained variance in the Rasch factor; ^b^Eigenvalue in the first contrast <3.0; ^c^ < 10% unexplained variance in the first contrast.

**Table 17:** SF-36 mental health scale Rasch analysis of summary of category structure for six waves of data collection.

	**WAVE 1**						**WAVE 2 (p. 120)**						**WAVE 3 (p. 125)**					
**CAT. LABEL**	**N**	**%**	**Average Measures**	**Infit MnSq**	**Outfit MnSq**	**Andrich Threshold**	**N**	**%**	**Average Measures**	**Infit MnSq**	**Outfit MnSq**	**Andrich Threshold**	**N**	**%**	**Average Measures**	**Infit MnSq**	**Outfit MnSq**	**Andrich Threshold**
1	22667	14	( -3.07)	*1.38*	1.20	NONE	18463	13	( -3.18)	*1.41*	1.22	NONE	14323	12	( -3.18)	*1.38*	1.22	NONE
2	49420	30	-1.06	.75	.78	-1.91	43019	30	-1.08	.76	.81	-2.03	33416	28	-1.12	.78	.85	-2.02
3	15086	9	-.17	.96	.86	.66	12291	8	-.15	.97	.89	.71	10845	9	-.17	.98	.85	.57
4	25646	15	.40	1.02	1.11	-.41^b^	20753	14	.43	1.00	1.12	-.38^b^	18002	15	.44	1.00	1.06	-.38^b^
5	28636	17	1.14	1.06	1.31	.51^b^	24231	17	1.16	1.08	*1.38*	.53^b^	18787	16	1.20	1.13	1.28	.63
6	24973	15	(2.54)	1.00	1.07	1.15	23360	16	(2.56)	1.00	1.08	1.17	18313	15	(2.60)	.95	1.02	1.19

	**WAVE 4**						**WAVE 5**						**WAVE 6**					
**CAT. LABEL**	**N**	**%**	**Average Measures**	**Infit MnSq**	**Outfit MnSq**	**Andrich Threshold**	**N**	**%**	**Average Measures**	**Infit MnSq**	**Outfit MnSq**	**Andrich Threshold**	**N**	**%**	**Average Measures**	**Infit MnSq**	**Outfit MnSq**	**Andrich Threshold**

1	12561	13	( -3.08)	*1.37*	1.21	NONE	10333	14	( -3.00)	*1.38*	1.23	NONE	7471	14	( -2.98)	*1.37*	1.21	NONE
2	27233	29	-1.11	.80	.88	-1.91	20854	28	-1.08	.82	.89	-1.82	14529	27	-1.09	.83	.91	-1.80
3	9548	10	-.18	.98	.82	.51	7515	10	-.19	.94	.76	.50	5675	11	-.19	.94	.78	.43
4	15240	16	.42	1.00	1.00	-.36^b^	12348	17	.40	.97	.94	-.40^b^	9024	17	.41	.98	.94	-.35^b^
5	15741	16	1.17	1.14	1.22	.60	12183	16	1.15	1.19	1.27	.61	8698	16	1.15	1.19	1.24	.64
6	15147	16	(2.57)	.93	.99	1.16	11454	15	(2.53)	.90	.99	1.11	8415	16	(2.51)	.90	.96	1.07

*Notes*. ^a^Andrich threshold category increase of >5; ^b^Andrich threshold category decrease where an increase is expected; values in italic for Infit or Outfit MnSq > 1.34; values underlined for Infit or Outfit MnSq < 0.64.

**Table 18:** Differential Item Functioning (DIF) for SF-36 mental health scale Rasch analysis for six waves of data collection based on marital status and area of residence.

	**Wave 1**				**Wave 2 **				**Wave 3**			
	**Marital status **		**Urban and Regional **		**Marital status **		**Urban and Regional **		**Marital status **		**Urban and Regional **	
**SF36 ITEM ** ** No.**	**SUMMARY DIF CHI-SQUARE (DIF = 2)**	**PROB.**	**DIF CONTRAST**	**Mantel- Haenszel Probability**	**SUMMARY DIF ** ** CHI-SQUARE (DIF = 2)**	**PROB.**	**DIF CONTRAST**	**Mantel- Haenszel Probability**	**SUMMARY DIF ** ** CHI-SQUARE (DIF = 2)**	**PROB.**	**DIF CONTRAST**	**Mantel- Haenszel Probability**
**17:Q5A**	3.13	.206	0.00	.720	0.05	.975	-0.17	.122	0.07	.969	0.00	.978
**18:Q5B**	6.16	.045^*∗*^	0.00	.799	0.41	.814	0.00	.347	0.59	.745	0.00	.165
**19:Q5C**	4.23	.119	0.00	.505	0.17	.922	-0.30	.066	0.79	.673	0.00	.484
**20:Q6**	62.55	.001^*∗∗∗*^	-0.09	.058	6.62	.036^*∗*^	0.00	.056	0.00	1.000	0.00	.415
**23:Q9A**	8.45	.014^*∗*^	0.00	.101	0.00	1.000	-0.05	.498	0.05	.979	0.00	.725
**24:Q9B**	14.83	.001^*∗∗∗*^	0.09	.001^*∗∗∗*^	0.41	.813	0.00	.553	11.22	.004^*∗∗*^	0.02	.093
**25:Q9C**	62.48	.001^*∗∗∗*^	0.12	.001^*∗∗∗*^	29.94	.001^*∗∗∗*^	0.48	.087	0.01	.996	0.07	.009^*∗∗*^
**26:Q9D**	9.01	.011^*∗*^	-0.07	.001^*∗∗∗*^	0.16	.925	-0.07	.476	22.89	.001^*∗∗∗*^	-0.06	.001^*∗∗∗*^
**27:Q9E**	8.72	.013^*∗*^	0.00	.741	0.49	.782	0.37	.010^*∗*^	0.57	.750	0.00	.207
**28:Q9F**	17.18	.001^*∗∗∗*^	0.08	.001^*∗∗∗*^	20.01	.001^*∗∗∗*^	0.00	.401	0.73	.694	0.05	.004
**29:Q9G**	5.04	.079	0.00	.719	3.76	.150	0.52	.003^*∗∗*^	11.42	.003^*∗∗*^	0.00	.815
**30:Q9H**	13.46	.001^*∗∗∗*^	-0.06	.001^*∗∗*^	8.62	.013^*∗*^	0.00	.176	3.70	.155	-0.07	.002^*∗∗*^
**31:Q9I**	1.75	.414	0.00	.224	0.51	.773	-0.18	.308	0.00	1.000	0.00	.299
**32:Q10**	14.70	.001^*∗∗∗*^	0.00	.207	0.11	.947	0.00	.165	2.75	.250	0.00	.978

	**Wave 4**				**Wave 5**				**Wave 6**			
**SF36 ITEM** **No.**	**Marital status**		**Urban and Regional**		**Marital status**		**Urban and Regional**		**Marital status**		**Urban and Regional**	
	**SUMMARY DIF** **CHI-SQUARE (DIF = 1)**	**PROB.**	**DIF CONTRAST**	**Mantel- Haenszel Probability**	**SUMMARY DIF** **CHI-SQUARE (DIF = 2)**	**PROB.**	**DIF CONTRAST**	**Mantel- Haenszel Probability**	**SUMMARY DIF** **CHI-SQUARE (DIF = 2)**	**PROB.**	**DIF CONTRAST**	**Mantel- Haenszel Probability**

**17:Q5A**	0.00	1.000	0.00	.618	0.07	.966	-0.03	.986	0.94	.623	-0.18	.001^*∗∗∗*^
**18:Q5B**	0.00	1.000	0.00	.639	2.43	.294	0.00	.395	0.39	.824	0.00	.543
**19:Q5C**	0.00	1.000	0.00	.497	0.71	.701	0.20	.271	0.59	.744	-0.19	.003^*∗∗*^
**20:Q6**	0.00	1.000	0.00	.779	6.37	.040^*∗*^	-0.02	.337	2.26	.320	-0.03	.162
**23:Q9A**	0.00	1.000	0.00	.900	1.95	.373	0.19	.254	1.18	.551	-0.04	.176
**24:Q9B**	6.95	.008^*∗∗*^	0.00	.384	13.76	.001^*∗∗∗*^	0.00	.784	3.06	.213	0.00	.580
**25:Q9C**	0.00	1.000	0.06	.030^*∗*^	6.84	.032^*∗*^	-0.68	.078	0.77	.678	0.08	.371
**26:Q9D**	0.00	1.000	0.00	.544	13.70	.001^*∗∗∗*^	-0.02	.118	2.06	.354	0.00	.923
**27:Q9E**	0.00	1.000	0.00	.537	3.30	.189	-0.08	.720	1.67	.430	0.06	.215
**28:Q9F**	0.00	1.000	0.00	.687	0.87	.644	0.00	.819	0.43	.806	0.00	.408
**29:Q9G**	0.00	1.000	0.00	.694	0.20	.908	0.27	.570	0.63	.729	0.03	.278
**30:Q9H**	6.10	.014^*∗*^	0.00	.297	4.86	.086	0.05	.065	0.08	.962	0.00	.419
**31:Q9I**	0.00	1.000	-0.05	.112	1.10	.574	0.48	.170	0.04	.981	-0.04	.664
**32:Q10**	0.00	1.000	0.00	.414	1.56	.456	0.08	.019^*∗*^	0.05	.979	0.00	.434

*Notes*. PROB. = probability; ^*∗*^*p* ≤ .05; ^*∗∗*^*p* ≤ .01; ^*∗∗∗*^*p* ≤ .001.

## Data Availability

The survey data used to support the findings of this study were supplied by the Data Access Committee of Australian Longitudinal Study on Women's Health by formal request. Requests for access to these data should be made to Data Access Committee of Australian Longitudinal Study on Women's Health.

## Supplementary Materials

Supplementary Materials One supplementary file submitted containing the below figures: Supplemental Figure 1: SF36 total scale Rasch analysis Person—item map for six waves of data collection. Supplemental Figure 2. Supplemental Figure 3: SF36 physical health scale Rasch analysis Person—item map for six waves of data collection. Supplemental Figure 4. Supplemental Figure 5: SF36 mental health scale Rasch analysis Person—item map for six waves of data collection. Supplemental Figure 6.
