# Supplementary material for: Evaluating the Longitudinal Item and Category Stability of the SF-36 Full and Summary Scales Using Rasch Analysis
Source: Biomed Res Int. 2018 Nov 4;2018:1013453. doi: 10.1155/2018/1013453 (PMC6241362; doi:10.1155/2018/1013453)
Supplement: Supplementary Materials — One supplementary file submitted containing the below figures: Supplemental Figure 1: SF36 total scale Rasch analysis Person—item map for six waves of data collection. Supplemental Figure 2. Supplemental Figure 3: SF36 physical health scale Rasch analysis Person—item map for six waves of data collection. Supplemental Figure 4. Supplemental Figure 5: SF36 mental health scale Rasch analysis Person—item map for six waves of data collection. Supplemental Figure 6. [file 1013453.f1.docx]

**Supplemental Figure 1: SF-36 total scale Rasch analysis Person – Item Map for six waves of data collection**

| **Wave 1** | **Wave 2** | **Wave 3** | **Wave 4** | **Wave 5** | **Wave 6** |
| --- | --- | --- | --- | --- | --- |
| SF-36 PERSON-ITEM MAP  <more>\|<rare>  2 + PF1  \|  \| RP2  \| RP3 \| RP4  \| RP1  \|S RE2 \| SF1  1 + RE1 \| RE3  . \| PF4  \| BP2  . \| PF6 \| PF7  . \| GH3 \| PF2 \| PF3  . \| PF5 \| MH5  0 . +M GH5 \| PF8  . T\| PF9  .## \| GH1 \| HT \| PF10 \| BP1 \| MH3  .########## S\|  .############ M\| GH4 \| VT1  .####### \| VT2  -1 .### S+  .# T\|S VT4  . \| SF2 \| GH2  . \|  . \| VT3  . \|  -2 . + MH1 \| MH4  . \|T  . \|  . \|  . \| MH2  \|  -3 +  \|  . \|  \|  \|  \|  -4 +  . \|  . \|  \|  \|  \|  -5 . +  <less>\|<frequ>  EACH '#' IS 306. | SF-36 PERSON-ITEM MAP  <more>\|<rare>  2 + PF1  \| RP2  \| RP3 \| RP4  \| RP1 \| SF1  \|S RE2  1 + RE1 \| RE3  \| PF4 \| BP2  \| PF6 \| PF7  \| PF2 \| PF3 \| MH5 \| GH3  . \| PF5  0 . +M PF8 \| GH5  . T\| PF9 \| BP1 \| MH3  .#### S\| GH1 \| HT \| PF10  ############# M\| VT1  .######## \| VT2 \| GH4  -1 .### S+  . T\|S VT4  . \| SF2 \| GH2  . \|  . \| VT3  -2 . +  . \| MH1 \| MH4  \|T  . \|  . \|  -3 + MH2  <less>\|<frequ>  EACH '#' IS 330. | SF-36 PERSON-ITEM MAP  <more>\|<rare>  2 + PF1  \| RP2  \| RP3 \| RP4  \| RP1  \|S RE2  1 + PF4 \| RE1 \| RE3 \| SF1  \| PF6 \| PF7  . \| PF2  \| PF3 \| BP2 \| GH3  . \| PF5 \| PF8 \| MH5  0 . +M PF9 \| GH5  . T\| PF10 \| MH3  .#### S\| GH1 \| BP1  .############ \| HT \| GH4  .########## M\| VT1  -1 .#### S+ VT2  .# T\|S VT4  . \| SF2 \| GH2  . \| VT3  . \|  -2 . +  . \| MH4  . \|T MH1  . \|  \|  -3 + MH2  <less>\|<frequ>  EACH '#' IS 246. | SF-36 PERSON-ITEM MAP  <more>\|<rare>  2 + PF1  \| RP2 \| RP4  \| RP3  \| RP1  \|S RE2  1 + PF4 \| RE1 \| RE3  \| PF6 \| PF7  . \| PF2 \| SF1  \| PF3 \| PF8  . \| PF5 \| BP2 \| MH5 \| GH3  0 . +M PF9  . T\| PF10 \| MH3 \| GH5  .## \|  .############ S\| GH1 \| HT \| BP1 \| GH4  .########### M\| VT1  -1 .##### S+ VT2 \| VT4  .# T\|S SF2  . \| GH2  . \| VT3  . \|  -2 . +  . \| MH4  . \|T MH1  . \|  \|  -3 + MH2  <less>\|<frequ>  EACH '#' IS 195. | SF-36 PERSON-ITEM MAP  <more>\|<rare>  2 + RP2 \| PF1  \| RP3 \| RP4  \|  \| RP1  \|S RE2 \| PF4  1 + RE1 \| RE3 \| PF7  \| PF2 \| PF6  \|  \| SF1 \| PF3 \| PF5 \| PF8  \| BP2 \| MH5  0 . +M GH3 \| PF9  . \| PF10  .## T\| MH3 \| GH5  .########### S\| BP1 \| GH4 \| GH1 \| HT  .############ M\|  -1 .####### S+ VT1 \| VT4  .## T\|S VT2 \| SF2  . \| GH2  . \| VT3  . \|  -2 . +  . \| MH4  \|T MH1  . \|  \| MH2  -3 +  <less>\|<frequ>  EACH '#' IS 149. | SF-36 PERSON-ITEM MAP  <more>\|<rare>  3 +  \|  \|T  \| PF1  \|  2 + RP2  \| RP3 \| RP4  \|  \| RP1  \|S PF4 \| PF7 \| RE2  1 + PF2 \| RE3  \| PF6 \| RE1  \| PF3 \| PF8  \| PF5 \| SF1  . \| PF9  0 . +M BP2 \| MH5 \| GH3  . \| PF10  .# T\| MH3 \| GH4 \| GH5  .####### S\| GH1 \| BP1  .############ M\| HT  -1 .######### S+ VT1 \| VT4  .### \|S SF2  . T\| VT2  . \| VT3 \| GH2  . \|  -2 . +  . \| MH4  . \| MH1  . \|T  . \| MH2  -3 +  <less>\|<frequ>  EACH '#' IS 111. |

*Physical functioning (PF) 1: vigorous activities limited; PF2: moderate activities limited; PF3: lifting or carrying; PF4: climbing several flights of stairs limited; PF5: climbing one flight of stairs limited; PF6: bending, kneeling, or stooping limited; PF7: walking more than one kilometre limited; PF8: walking half kilometre limited; PF9: walking 100 metres limited; PF10: bathing or dressing limited*

*Role-Physical (RP) 1: cut down time spent on work/activity; RP2: accomplish less; RP3: limited in type of work; RP4: difficulty performing*

*Bodily Pain (BP) 1: intensity of bodily pain; BP2: pain interfered with work*

*General health (GH) 1: general health; GH2: get sick easier than other people; GH3: as healthy as anybody I know; GH4: expect my health to get worse; GH5: my health is excellent*

*Vitality (VT) 1: feel full of life; VT2: have a lot of energy; VT3: feel worn out; VT4: feel tired*

*Social functioning (SF) 1: health interfere with social activities; SF2: how much time health problems interfere with social activities*

*Role-Emotional (RE) 1: cut down time spent on work/activity; RE2: accomplish less; RE3: less careful at work*

*Mental Health (MH) 1: felt nervous; MH2: felt down in dumps; MH3: felt calm and peaceful; MH4: felt down; MH5: felt happy*

*Reported Health Transition (HT): Health now compared to 1 year ago*

**Supplemental Figure 2: SF-36 total scale Rasch analysis Category Probabilities for six waves of data collection**

| WAVE 1 CATEGORY PROBABILITIES: MODES - Structure measures at intersections (p.73)  P ++-------+-------+-------+-------+-------+-------+-------++  R 1.0 + +  O \| \|  B \|11 \|  A \| 111 \|  B .8 + 11 66+  I \| 11 66 \|  L \| 11 6 \|  I \| 1 66 \|  T .6 + 11 6 +  Y \| 1 66 \|  .5 + 1 6 +  O \| 1*22222222 6 \|  F .4 + 222 1 22 333 55555*55 +  \| 22 1 33* 333 55 6 555 \|  R \| 22 11 33 22 35 66 55 \|  E \| 22 *3 2 4*** 6 55 \|  S .2 + 222 33 11 4**5 **44 555+  P \| 222 33 11 44 5 2 6633 44 \|  O \|22 333 4** 55 ** 33 4444 \|  N \| 333333 44444555*11*66 2222 3333 44444 \|  S .0 +**************************666666 11111111****************+  E ++-------+-------+-------+-------+-------+-------+-------++  -4 -3 -2 -1 0 1 2 3  PERSON [MINUS] ITEM MEASURE | WAVE 2 CATEGORY PROBABILITIES: MODES - Structure measures at intersections  P ++-------+-------+-------+-------+-------+-------+-------++  R 1.0 + +  O \| \|  B \|1 \|  A \| 111 \|  B .8 + 111 6+  I \| 11 66 \|  L \| 1 66 \|  I \| 11 66 \|  T .6 + 1 6 +  Y \| 11 6 \|  .5 + 1 22 66 +  O \| *222 2222 6 \|  F .4 + 22 11 2233333 55555*555 +  \| 22 1 332 33 5 6 55 \|  R \| 22 1 33 22 355 6 55 \|  E \| 222 1*3 2 4***4 66 555 \|  S .2 + 22 33 11 4**5 3*44 55+  P \| 222 33 11 44 5 2 6633 444 \|  O \|22 333 4** 55 ** 33 444 \|  N \| 333333 44444555*11*66 2222 3333 444444 \|  S .0 +**************************666666 11111111****************+  E ++-------+-------+-------+-------+-------+-------+-------++  -4 -3 -2 -1 0 1 2 3  PERSON [MINUS] ITEM MEASURE |
| --- | --- |
| Wave 3 CATEGORY PROBABILITIES: MODES - Structure measures at intersections  P ++-------+-------+-------+-------+-------+-------+-------++  R 1.0 + +  O \| \|  B \|11 \|  A \| 111 \|  B .8 + 11 66+  I \| 11 6 \|  L \| 11 66 \|  I \| 1 66 \|  T .6 + 11 6 +  Y \| 1 6 \|  .5 + 1 66 +  O \| 1*2222222 6 \|  F .4 + 222 1 22 33 5555*55 +  \| 22 1 3** 333 55 6 555 \|  R \| 22 11 33 2 3355 6 55 \|  E \| 22 *3 22 44**44466 555 \|  S .2 + 222 33 11 44*55 336444 55+  P \| 222 33 11 44 522 6633 44 \|  O \|22 333 44*1 55 ** 33 444 \|  N \| 333333 44444 555*11*66 2222 3333 444444 \|  S .0 +**************************666666 11111111****************+  E ++-------+-------+-------+-------+-------+-------+-------++  -4 -3 -2 -1 0 1 2 3  PERSON [MINUS] ITEM MEASURE | WAVE 4 CATEGORY PROBABILITIES: MODES - Structure measures at intersections  P ++-------+-------+-------+-------+-------+-------+-------++  R 1.0 + +  O \| \|  B \|11 \|  A \| 111 \|  B .8 + 111 66+  I \| 11 66 \|  L \| 1 6 \|  I \| 11 66 \|  T .6 + 1 6 +  Y \| 11 6 \|  .5 + 1 66 +  O \| 12222222 6 \|  F .4 + 22211 22 55555*55 +  \| 22 1 **3333 55 6 555 \|  R \| 22 1 333 2 33 5 6 55 \|  E \| 22 1* 22444**44466 55 \|  S .2 + 222 33 1 44255 336444 555+  P \| 222 333 11 44 5522 663 44 \|  O \|222 333 44*155 ** 333 444 \|  N \| 33333 44444 555111*66 2222 3333 444444 \|  S .0 +**************************666666 11111111****************+  E ++-------+-------+-------+-------+-------+-------+-------++  -4 -3 -2 -1 0 1 2 3  PERSON [MINUS] ITEM MEASURE |
| Wave 5 CATEGORY PROBABILITIES: MODES - Structure measures at intersections  P ++-------+-------+-------+-------+-------+-------+-------++  R 1.0 + +  O \| \|  B \|111 \|  A \| 111 \|  B .8 + 11 66+  I \| 11 66 \|  L \| 11 66 \|  I \| 1 6 \|  T .6 + 11 6 +  Y \| 1 66 \|  .5 + 11 6 +  O \| 1222222 6 \|  F .4 + 2221 22 5555*55 +  \| 22 1 2*333 55 6 55 \|  R \| 222 11 333 2 33*4** 66 555 \|  E \| 22 *3 2*44 * 44* 55 \|  S .2 + 222 33 11 44 255 33 6 44 555+  P \| 222 33 144 5522 6*3 44 \|  O \|222 333 4411*5 ** 33 444 \|  N \| 333333 44444 555 1***6 222 3333 444444 \|  S .0 +**************************66666 111111*****************+  E ++-------+-------+-------+-------+-------+-------+-------++  -4 -3 -2 -1 0 1 2 3  PERSON [MINUS] ITEM MEASURE | Wave 6 CATEGORY PROBABILITIES: MODES - Structure measures at intersections  P ++---------+---------+---------+---------+---------+---------++  R 1.0 + +  O \| \|  B \| \|  A \| \|  B .8 +1 666+  I \| 11 66 \|  L \| 11 66 \|  I \| 11 66 \|  T .6 + 11 66 +  Y \| 11 6 \|  .5 + 1 66 +  O \| 112222222 6 \|  F .4 + 22221 222 5555**5 +  \| 222 11 2*3333 555 6 5555 \|  R \| 22 11 3333 22 33*44** 66 555 \|  E \| 222 *3 2444 ** 444* 555 \|  S .2 +22 333 11 4442255 33 66 444 555 +  P \| 333 1144 5522 6*33 444 5\|  O \| 333 444411155 *** 33 444 \|  N \| 3333333 44444 5555511***6 2222 333333 4444444 \|  S .0 +**********************6666666 11111111*********************+  E ++---------+---------+---------+---------+---------+---------++  -3 -2 -1 0 1 2 3  PERSON [MINUS] ITEM MEASURE |

**Supplemental Figure 3: SF-36 physical health scale Rasch analysis Person – Item Map for six waves of data collection**

| **Wave 1** | **Wave 2** | **Wave 3** | **Wave 4** | **Wave 5** | **Wave 6** |
| --- | --- | --- | --- | --- | --- |
| SF-36 PERSON-ITEM MAP  <more>\|<rare>  2 +  \|T  . \| PF1  \| RP2  \| RP3 \| RP4  1 . +S RP1  \|  \| PF4  . \| BP2  \| PF6 \| PF7  0 . +M GH3 \| PF2 \| PF3  \| PF5  . \| GH5 \| PF8  . \| PF9  . \| GH1 \| PF10 \| BP1  -1 . T+S  .# \| GH4  .########## S\|  .############ \|  .####### M\|T  -2 .######## +  .##### S\| GH2  .## \|  .# T\|  . \|  -3 . +  . \|  . \|  . \|  . \|  -4 . +  . \|  . \|  \|  . \|  -5 +  . \|  \|  \|  \|  -6 . +  <less>\|<frequ>  EACH '#' IS 243. | SF-36 PERSON-ITEM MAP  <more>\|<rare>  2 +T  \|  \| PF1  \| RP2  \| RP3 \| RP4  1 +S RP1 \| BP1  \|  \|  \| PF4  \| PF6 \| PF7  0 +M PF2 \| PF3 \| GH3  \| PF5  . \| PF8 \| GH5  \| PF9  . \| PF10 \| BP2  -1 . +S GH1  .# T\|  .###### S\| GH4  .############ \|  .############ M\|  -2 .###### +T  .###### S\|  .### \| GH2  .# T\|  . \|  -3 . +  . \|  . \|  . \|  . \|  -4 . +  . \|  \|  \|  \|  -5 +  <less>\|<frequ>  EACH '#' IS 197. | SF-36 PERSON-ITEM MAP  <more>\|<rare>  2 +T  \|  \| PF1  \| RP2  \| RP3 \| RP4  1 +S RP1  \|  \| PF4  \| PF6 \| PF7  \| PF2  0 . +M PF3 \| BP2 \| GH3  \| PF5 \| PF8  \| GH5  . \| PF9  . \| PF10  -1 . +S GH1 \| BP1  .# T\| GH4  .###### \|  .############ S\|  .############ \|  -2 .######## M+T  .######## \|  .### S\| GH2  .## \|  . T\|  -3 . +  . \|  . \|  . \|  . \|  -4 . +  . \|  \|  . \|  . \|  -5 +  . \|  \|  \|  \|  -6 . +  <less>\|<freq>  EACH "#" IS 144: EACH "." IS 1 TO 143 | SF-36 PERSON-ITEM MAP  <more>\|<rare>  2 +T  \|  \| PF1  \| RP2 \| RP4  \| RP3  1 . +S RP1  \|  \| PF4  \| PF6 \| PF7  \| PF2  0 +M PF3 \| PF8  \| PF5 \| BP2 \| GH3  \| PF9  . \| GH5  . \| PF10  -1 . +S GH1 \| BP1  .# T\| GH4  .##### \|  .########### S\|  .############ \|  -2 .############ M+T  .########## \|  .#### S\| GH2  .## T\|  . \|  -3 . +  . \|  . \|  . \|  . \|  -4 . +  . \|  . \|  . \|  \|  -5 . +  \|  \|  \|  \|  -6 . +  <less>\|<frequ>  EACH '#' IS 113. | SF-36 PERSON-ITEM MAP  <more>\|<rare>  2 +T  \|  \| PF1  \| RP2 \| RP4  \| RP3  1 +S RP1  \|  \| PF4 \| PF7  \| PF2  \| PF6  0 +M PF3 \| PF5 \| PF8  \|  \| BP2 \| GH3 \| PF9  . \|  . \| GH5 \| PF10  -1 . +S GH4  .# T\| BP1 \| GH1  .##### \|  .########## S\|  .############ \|  -2 .############ M+T  .######## \|  .####### S\| GH2  .## \|  . T\|  -3 . +  . \|  . \|  . \|  . \|  -4 . +  . \|  . \|  . \|  \|  -5 +  <less>\|<frequ>  EACH '#' IS 89. | SF-36 PERSON-ITEM MAP  <more>\|<rare>  2 +  \| PF1  \|  \| RP2 \| RP4  \| RP3  1 +S RP1  \| PF4 \| PF7  \|  \| PF2 \| PF6  \| PF8  0 +M PF3 \| PF5  \|  \| PF9  \| BP2 \| GH3  . \| PF10  -1 . +S GH4 \| GH5  . T\|  .## \| GH1 \| BP1  .####### S\|  .######### \|  -2 .############ M+  .############ \|T  .####### S\| GH2  .##### \|  .# T\|  -3 . +  . \|  . \|  . \|  . \|  -4 . +  . \|  . \|  \|  \|  -5 +  \|  \|  \|  \|  -6 . +  <less>\|<freq>  EACH "#" IS 66: EACH "." IS 1 TO 65 |

Physical functioning (PF) 1: vigorous activities limited; PF2: moderate activities limited; PF3: lifting or carrying; PF4: climbing several flights of stairs limited; PF5: climbing one flight of stairs limited; PF6: bending, kneeling, or stooping limited; PF7: walking more than one kilometre limited; PF8: walking half kilometre limited; PF9: walking 100 metres limited; PF10: bathing or dressing limited

Role-Physical (RP) 1: cut down time spent on work/activity; RP2: accomplish less; RP3: limited in type of work; RP4: difficulty performing

Bodily Pain (BP) 1: intensity of bodily pain; BP2: pain interfered with work

General health (GH) 1: general health; GH2: get sick easier than other people; GH3: as healthy as anybody I know; GH4: expect my health to get worse; GH5: my health is excellent

**Supplemental Figure 4: SF-36 physical health scale Rasch analysis Category Probabilities for six waves of data collection**

| WAVE 1 CATEGORY PROBABILITIES: MODES - Structure measures at intersections  P ++-----+-----+-----+-----+-----+-----+-----+-----+-----+-----++  R 1.0 + +  O \| \|  B \| \|  A \| 55555 66\|  B .8 +1 55 555 66 +  I \| 11 55 55 6 \|  L \| 1 5 5 66 \|  I \| 1 5 55 6 \|  T .6 + 1 5 5 6 +  Y \| 1 5 5 66 \|  .5 + 1 333 5 * +  O \| 122222 33 33 5 6 55 \|  F .4 + 221 2* 35 6 5 +  \| 22 1 3 2 53 66 5 \|  R \| 2 133 22 5 3 6 55 \|  E \| 22 31 2 5 3 6 5 \|  S .2 +22 3 1 25 4444* 66 55 +  P \| 33 11 *** *44 666 55\|  O \| 33 1*4* 2 334444666 \|  N \| 3333 444*5*11 2222 6****444444 \|  S .0 +****************66666****************************************+  E ++-----+-----+-----+-----+-----+-----+-----+-----+-----+-----++  -4 -3 -2 -1 0 1 2 3 4 5 6  PERSON [MINUS] ITEM MEASURE | WAVE 2 CATEGORY PROBABILITIES: MODES - Structure measures at intersections  P ++-----+-----+-----+-----+-----+-----+-----+-----+-----+-----++  R 1.0 + +  O \| \|  B \| \|  A \| 555 66\|  B .8 +1 555 5555 66 +  I \| 1 5 55 6 \|  L \| 11 5 5 66 \|  I \| 1 5 55 6 \|  T .6 + 1 5 5 6 +  Y \| 1 5 5 6 \|  .5 + 1 33333 5 *6 +  O \| 12222 33 3 5 6 55 \|  F .4 + 221 2*2 35 6 5 +  \| 22 1 3 2 * 6 5 \|  R \| 22 13 2 5 3 66 55 \|  E \| 2 31 22 5 3 6 5 \|  S .2 +22 33 1 2544444*4 66 55 +  P \| 3 1 4** 3*44 66 55\|  O \| 33 11445 22 3 4444666 \|  N \| 3333 444***11 222 6****6444444 \|  S .0 +****************66666****************************************+  E ++-----+-----+-----+-----+-----+-----+-----+-----+-----+-----++  -4 -3 -2 -1 0 1 2 3 4 5 6  PERSON [MINUS] ITEM MEASURE |
| --- | --- |
| WAVE 3 Physical CATEGORY PROBABILITIES: MODES - Andrich thresholds at intersections  P -+-----+-----+-----+-----+-----+-----+-----+-----+-----+-----+-  R 1.0 + +  O \| \|  B \| \|  A \| 55555 6\|  B .8 +1 55 555 66 +  I \| 11 55 55 66 \|  L \| 1 5 55 6 \|  I \| 1 5 5 6 \|  T .6 + 1 5 55 66 +  Y \| 1 5 5 6 \|  .5 + 1 33 5 * +  O \| 1 222 33 33 5 6 5 \|  F .4 + 22* 223 3 5 66 55 +  \| 2 1 3322 * 6 5 \|  R \| 22 13 2 53 6 5 \|  E \| 22 31 2 5 3 66 55 \|  S .2 +22 3 1 22*4444**4 66 55 +  P \| 33 11 4*2 3444 66 5\|  O \| 33 ***5 22 33 4444666 \|  N \| 3333 444455111 222 6***66444444 \|  S .0 +****************66666****************************************+  E -+-----+-----+-----+-----+-----+-----+-----+-----+-----+-----+-  -4 -3 -2 -1 0 1 2 3 4 5 6  PERSON [MINUS] ITEM MEASURE | WAVE 4 CATEGORY PROBABILITIES: MODES - Structure measures at intersections  P ++-----+-----+-----+-----+-----+-----+-----+-----+-----+-----++  R 1.0 + +  O \| \|  B \| \|  A \| 5 66\|  B .8 +11 5555 5555 66 +  I \| 1 5 55 66 \|  L \| 11 55 55 6 \|  I \| 1 5 5 6 \|  T .6 + 1 5 55 66 +  Y \| 1 5 5 6 \|  .5 + 1 5 * +  O \| 1 3333 5 6 5 \|  F .4 + 22*222233 33 5 6 55 +  \| 22 1 322 * 66 5 \|  R \| 2 13 2 53 6 5 \|  E \| 22 31 22 544*4 66 55 \|  S .2 + 22 33 1 **4 3444 6 55 +  P \|2 3 1 4452 33 444 666 55\|  O \| 333 **55 22 33 44*66 \|  N \| 3333 444455111 222 6***6 444444 \|  S .0 +****************66666****************************************+  E ++-----+-----+-----+-----+-----+-----+-----+-----+-----+-----++  -4 -3 -2 -1 0 1 2 3 4 5 6  PERSON [MINUS] ITEM MEASURE |
| WAVE 5 CATEGORY PROBABILITIES: MODES - Structure measures at intersections  P ++-----+-----+-----+-----+-----+-----+-----+-----+-----+-----++  R 1.0 + +  O \| \|  B \| 6\|  A \|1 66 \|  B .8 + 1 5555555 66 +  I \| 11 55 55 66 \|  L \| 1 5 55 6 \|  I \| 1 5 5 6 \|  T .6 + 1 5 55 66 +  Y \| 1 5 5 6 \|  .5 + 1 5 * +  O \| 1 333 5 6 5 \|  F .4 + 2*2222233 3 5 6 55 +  \| 22 1 32 335 66 5 \|  R \| 22 133 22 53 6 5 \|  E \| 22 31 2 *44*44 66 55 \|  S .2 + 22 3 1 ** 3 444 6 55 +  P \|2 33 11 4452 33 44 666 55 \|  O \| 33 4*55 22 3 4**6 5\|  N \| 3333 4444551111 222 6**** 444444 \|  S .0 +****************666666***************************************+  E ++-----+-----+-----+-----+-----+-----+-----+-----+-----+-----++  -4 -3 -2 -1 0 1 2 3 4 5 6  PERSON [MINUS] ITEM MEASURE | WAVE 6 CATEGORY PROBABILITIES: MODES - Andrich thresholds at intersections  P -+-----+-----+-----+-----+-----+-----+-----+-----+-----+-----+-  R 1.0 + +  O \| \|  B \| \|  A \|1 66\|  B .8 + 11 555555555 66 +  I \| 1 5 55 66 \|  L \| 1 55 55 6 \|  I \| 1 5 5 66 \|  T .6 + 1 5 5 6 +  Y \| 1 5 55 6 \|  .5 + 1 5 * +  O \| 1 5 6 5 \|  F .4 + 2*2222 333333 5 66 5 +  \| 22 1 *2 35 6 55 \|  R \| 22 133 2 53 6 5 \|  E \| 22 31 22 4*4**44 66 55 \|  S .2 + 22 3 1 4*5 3 444 66 55 +  P \|2 33 11 4 52 3 44 66 55\|  O \| 33 44*55 22 33 44*66 \|  N \| 3333 444555111 222 6****6 444444 \|  S .0 +***************666666****************************************+  E -+-----+-----+-----+-----+-----+-----+-----+-----+-----+-----+-  -4 -3 -2 -1 0 1 2 3 4 5 6  PERSON [MINUS] ITEM MEASURE |

**Supplemental Figure 5: SF-36 mental health scale Rasch analysis Person – Item Map for six waves of data collection**

| **Wave 1** | **Wave 2** | **Wave 3** | **Wave 4** | **Wave 5** | **Wave 6** |
| --- | --- | --- | --- | --- | --- |
| SF-36 Person-Item Map  <more>\|<rare>  2 +  . \|  \|  . \| RE2 \| SF1  . \| RE3  . \| RE1  . \|S  1 . +  . \|  . \|  . T\| MH5  .# \|  .### S\|  .####### \| MH3  0 ############# +M VT1  .############ M\| VT2  .####### \|  .### S\|  .## \| VT4  . T\| SF2  . \| VT3  -1 . +  . \|S  . \| MH1 \| MH4  . \|  . \|  . \|  . \| MH2  -2 . +  . \|T  . \|  . \|  \|  \|  . \|  -3 +  \|  \|  \|  \|  . \|  \|  -4 . +  <less>\|<freq>  EACH "#" IS 224: EACH "." IS 1 TO 223 | SF-36 Person-Item Map  <more>\|<rare>  2 +  \| SF1  . \| RE2  \|  . \| RE1 \| RE3  . \|S  1 . +  . \|  . \| MH5  .# T\|  .#### \| MH3  .######## S\|  0 .########### M+M VT1  .############ \| VT2  .#### S\|  .# \| VT4  . T\| SF2  . \|  -1 . + VT3  . \|S  . \| MH4  . \| MH1  \|  . \|  -2 . + MH2  \|  . \|T  \|  . \|  \|  -3 +  \|  \|  . \|  \|  \|  -4 +  \|  \|  \|  \|  \|  -5 . +  <less>\|<frequ>  EACH '#' IS 224. | SF-36 Person-Item Map  <more>\|<rare>  4 . +  \|  \|  \|  \|  \|  3 +  \|  \|  \|  \|T  \|  2 +  \| RE2  \|  \| RE1 \| RE3  . \| SF1  . \|S  1 . +  . \|  . T\| MH5  .## \|  .#### S\| MH3  .######## \|  0 .########### M+M  .############ \| VT1  .### S\| VT2  .# \| VT4 \| SF2  . T\|  . \| VT3  -1 . +  . \|S  . \| MH4  . \| MH1  . \|  . \|  -2 . +  . \| MH2  . \|T  . \|  . \|  \|  -3 . +  \|  \|  \|  \|  \|  -4 +  \|  \|  \|  \|  \|  -5 . +  <less>\|<frequ>  EACH '#' IS 177. | SF-36 Person-Item Map  <more>\|<rare>  4 . +  \|  \|  \|  \|  \|  3 +  \|  \|  \|  . \|T  \|  2 +  . \| RE2  . \|  . \| RE1 \| RE3  . \|  . \|S SF1  1 . +  . \|  . \| MH5  .# T\|  .#### S\| MH3  .####### \|  0 .############ M+M  .############ \| VT1  #### S\| VT2 \| VT4  .## \| SF2  . T\|  . \| VT3  -1 . +  . \|S  . \| MH4  . \| MH1  . \|  . \|  -2 . + MH2  \|  . \|T  . \|  \|  \|  -3 +  . \|  \|  \|  \|  \|  -4 +  \|  \|  \|  \|  \|  -5 . +  <less>\|<freq>  EACH "#" IS 151: EACH "." IS 1 TO 150 | SF-36 Person-Item Map  <more>\|<rare>  4 . +  \|  \|  \|  \|  \|  3 +  \|  \|  \|  \|  . \|T  2 +  \| RE2  \|  \| RE1 \| RE3  \|  . \|S  1 . + SF1  . \|  . \| MH5  .# T\|  .## \| MH3  .######## S\|  0 .########## M+M  .############ \| VT1  .#### S\| VT2 \| VT4  .## \| SF2  . T\| VT3  . \|  -1 . +  . \|S  . \| MH4  . \| MH1  . \|  . \|  -2 . + MH2  . \|T  \|  \|  \|  \|  -3 . +  \|  \|  \|  \|  \|  -4 +  \|  \|  \|  \|  \|  -5 . +  <less>\|<freq>  EACH "#" IS 124: EACH "." IS 1 TO 123 | SF-36 Person-Item Map  <more>\|<rare>    3 +  \|  \|  \|  \|  \|T  . \|  2 +  \| RE2  \|  \| RE1 \| RE3  . \|  \|  \|S  1 . + SF1  . \|  . \| MH5  . T\|  .# \|  .#### S\| MH3  .######## \|  0 .############ M+M  .########### \|  .####### S\| VT1 \| VT4  .### \| SF2  ## \| VT2  . T\| VT3  . \|  -1 . +  . \|S  . \| MH4  . \| MH1  . \|  . \|  . \| MH2  -2 . +  \|  \|T  . \|  . \|  \|  . \|  -3 +  <less>\|<freq>  EACH "#" IS 71: EACH "." IS 1 TO 70 |

*Vitality (VT) 1: feel full of life; VT2: have a lot of energy; VT3: feel worn out; VT4: feel tired, Social functioning (SF) 1: health interfere with social activities; SF2: how much time health problems interfere with social activities*

*Role-Emotional (RE) 1: cut down time spent on work/activity; RE2: accomplish less; RE3: less careful at work, Mental Health (MH) 1: felt nervous; MH2: felt down in dumps; MH3: felt calm and peaceful; MH4: felt down; MH5: felt happy*

*Reported Health Transition (HT): Health now compared to 1 year ago*

**Supplemental Figure 6: SF-36 mental health scale Rasch analysis Category Probabilities for six waves of data collection**

| Wave 1 Mental health CATEGORY PROBABILITIES: MODES - Andrich thresholds at intersections  P -+-------+-------+-------+-------+-------+-------+-------+-  R 1.0 + +  O \| \|  B \|11 \|  A \| 111 66\|  B .8 + 11 66 +  I \| 11 66 \|  L \| 11 66 \|  I \| 11 6 \|  T .6 + 1 22 66 +  Y \| 11 2222 222 6 \|  .5 + 122 2 6 +  O \| 2211 22 6 \|  F .4 + 22 1 2 6 +  \| 2 11 2 555*5555 \|  R \| 22 1 244** 6 555 \|  E \| 222 11 44* 5 ** 55 \|  S .2 + 22 11 4 5*2 6 44 555 +  P \| 222 3***33*333* 444 555\|  O \|22 333*4411*5 663** 444 \|  N \| 333333*444 5555 ***1 ***33 44444 \|  S .0 +**************************66666 1111111***************+  E -+-------+-------+-------+-------+-------+-------+-------+-  -4 -3 -2 -1 0 1 2 3  PERSON [MINUS] ITEM MEASURE | WAVE 2 CATEGORY PROBABILITIES: MODES - Structure measures at intersections  P ++-------+-------+-------+-------+-------+-------+-------++  R 1.0 + +  O \| \|  B \|1 \|  A \| 111 66\|  B .8 + 11 66 +  I \| 11 66 \|  L \| 11 66 \|  I \| 11 6 \|  T .6 + 1 222222 6 +  Y \| 11 222 22 66 \|  .5 + 122 2 6 +  O \| 2211 2 6 \|  F .4 + 22 1 2 6 +  \| 2 11 2 555*5555 \|  R \| 22 1 244** 6 555 \|  E \| 222 11 4425 4*4 55 \|  S .2 + 22 11 44 552 66 44 555 +  P \| 222 ****33*333* 44 555\|  O \|2 33334411*5 663** 444 \|  N \| 333333*44445555 *** ***33 44444 \|  S .0 +**************************66666 11111111***************+  E ++-------+-------+-------+-------+-------+-------+-------++  -4 -3 -2 -1 0 1 2 3  PERSON [MINUS] ITEM MEASURE |
| --- | --- |
| WAVE 3 CATEGORY PROBABILITIES: MODES - Structure measures at intersections  P ++-------+-------+-------+-------+-------+-------+-------++  R 1.0 + +  O \| \|  B \|1 \|  A \| 111 66\|  B .8 + 11 66 +  I \| 11 66 \|  L \| 11 6 \|  I \| 11 66 \|  T .6 + 1 22222 6 +  Y \| 11 222 22 6 \|  .5 + 122 2 6 +  O \| 2211 2 66 \|  F .4 + 22 1 22 6 +  \| 2 11 2 55*5555 \|  R \| 22 1 24444** 6 555 \|  E \| 222 11 442 55 *4 55 \|  S .2 + 22 11 **33 *2 6 44 555 +  P \| 222 3*** 5*33*6 44 555\|  O \|2 333344 1155 66 **3 444 \|  N \| 33333334444 55551*** 2**33 444444 \|  S .0 +**************************66666 11111111***************+  E ++-------+-------+-------+-------+-------+-------+-------++  -4 -3 -2 -1 0 1 2 3  PERSON [MINUS] ITEM MEASURE | WAVE 4 CATEGORY PROBABILITIES: MODES - Andrich thresholds at intersections  P -+-------+-------+-------+-------+-------+-------+-------+-  R 1.0 + +  O \| \|  B \|11 \|  A \| 111 66\|  B .8 + 11 66 +  I \| 11 66 \|  L \| 11 66 \|  I \| 11 6 \|  T .6 + 1 6 +  Y \| 11 2222222 66 \|  .5 + 1222 22 6 +  O \| 2211 2 6 \|  F .4 + 22 1 2 6 +  \| 2 1 2 555*5555 \|  R \| 22 11 2444**4 6 555 \|  E \| 222 11 442 5 *4 55 \|  S .2 + 22 1 3**33** 66 44 555 +  P \| 222 33**4 5 3** 44 555\|  O \|22 333 44 11*5 66 *33 444 \|  N \| 33333334444 5555 *** 22**33 44444 \|  S .0 +**************************66666 11111111***************+  E -+-------+-------+-------+-------+-------+-------+-------+-  -4 -3 -2 -1 0 1 2 3  PERSON [MINUS] ITEM MEASURE |
| Wave 5 CATEGORY PROBABILITIES: MODES - Andrich thresholds at intersections  P -+---------+---------+---------+---------+---------+---------+-  R 1.0 + +  O \| \|  B \| \|  A \| 666\|  B .8 + 666 +  I \|11 66 \|  L \| 11 66 \|  I \| 111 66 \|  T .6 + 1 6 +  Y \| 11 2222222 66 \|  .5 + 112222 222 6 +  O \| 2211 2 6 \|  F .4 + 22 11 22 66 +  \| 222 1 2 555*55555 \|  R \| 22 11 24444***466 555 \|  E \|222 11 4442 55 644 555 \|  S .2 + 11 3**3333*2 66 444 5555 +  P \| 33*** 5533** 44 5555\|  O \| 33333444 1*55 666 **3 4444 \|  N \| 3333333344444 5555 ****1 2***333 444444 \|  S .0 +***********************66666 111111111*******************+  E -+---------+---------+---------+---------+---------+---------+-  -3 -2 -1 0 1 2 3  PERSON [MINUS] ITEM MEASURE | WAVE 6 CATEGORY PROBABILITIES: MODES - Andrich thresholds at intersections  P -+---------+---------+---------+---------+---------+---------+-  R 1.0 + +  O \| \|  B \| \|  A \| 666\|  B .8 + 666 +  I \|11 66 \|  L \| 111 66 \|  I \| 11 66 \|  T .6 + 11 6 +  Y \| 1 2222222 66 \|  .5 + 11 222 22 6 +  O \| 22*1 22 66 \|  F .4 + 22 11 2 6 +  \| 222 11 22 55*5555 \|  R \| 22 1 244444***66 5555 \|  E \|222 11 4442 55 644 555 \|  S .2 + 11 33**3333** 66 444 555 +  P \| 333*** 5533** 44 55555\|  O \| 3333 444 1**5 6662**3 4444 \|  N \| 3333333344444 5555 6***1 2***333 444444 \|  S .0 +***********************66666 111111111*******************+  E -+---------+---------+---------+---------+---------+---------+-  -3 -2 -1 0 1 2 3 |
